# Listeners’ baseline autonomic states associated with distinct music-physiology response patterns

**DOI:** 10.1093/ehjimp/qyag013

**Published:** 2026-01-19

**Authors:** Mateusz Soliński, Vanessa Pope, Pier Lambiase, Elaine Chew

**Affiliations:** School of Biomedical Engineering & Imaging Sciences, Faculty of Life Sciences & Medicine, King’s College London, WC2R 2LS, London, UK; Engineering Department, Faculty of Natural, Mathematical & Engineering Sciences, King’s College London, WC2R 2LS, London, UK; School of Biomedical Engineering & Imaging Sciences, Faculty of Life Sciences & Medicine, King’s College London, WC2R 2LS, London, UK; Engineering Department, Faculty of Natural, Mathematical & Engineering Sciences, King’s College London, WC2R 2LS, London, UK; School of Biomedical Engineering & Imaging Sciences, Faculty of Life Sciences & Medicine, King’s College London, WC2R 2LS, London, UK; Barts Heart Centre, St Bartholomew’s Hospital, West Smithfield, EC1A 7BE, London, UK; School of Biomedical Engineering & Imaging Sciences, Faculty of Life Sciences & Medicine, King’s College London, WC2R 2LS, London, UK; Engineering Department, Faculty of Natural, Mathematical & Engineering Sciences, King’s College London, WC2R 2LS, London, UK

**Keywords:** autonomic nervous system, digital music therapeutics, change points, canonical correlation analysis, interpretation map

## Abstract

**Aims:**

Personalizing music-based interventions requires an understanding of how individuals’ physiology responds to expressive music events. Although music can modulate autonomic function, variability across listeners has limited its therapeutic use. We introduce a change-point-driven framework that links music features to cardiovascular responses and test whether baseline autonomic balance is associated with group- and individual-level response patterns.

**Methods and results:**

Physiological signals from 112 participants (63 females, 21–79 years, including 25 with elevated resting blood pressure) collected whilst they listened to nine expressive versions of eight pieces of Western classical music were analysed using canonical correlation analysis. Listeners were stratified by their baseline sympathetic–parasympathetic balance. The first two canonical variates identified the dominant links between music and physiologic change points. We developed a new method for visually representing these couplings, termed change point connectivity graphs. Listeners with high parasympathetic tone showed greater vagal engagement in response to novel melodies and increased acoustic intensity, whereas those with elevated sympathetic drive exhibited amplified sympathetic responses. Novelty was the most consistent musical driver of autonomic change.

**Conclusion:**

These findings demonstrate that baseline autonomic activity might determine whether expressive music changes elicit parasympathetic engagement or sympathetic activation. The change point connectivity graphs open new pathways to selecting or controlling expressive music structures to support the achievement of targeted physiologic effects. Our framework is a step towards precision music therapeutics by enabling the selection or modulation of music features to produce autonomic effects for cardiovascular prevention, rehabilitation, and practice, including applications via wearable technologies.

## Graphical Abstract

**Graphical Abstract:**
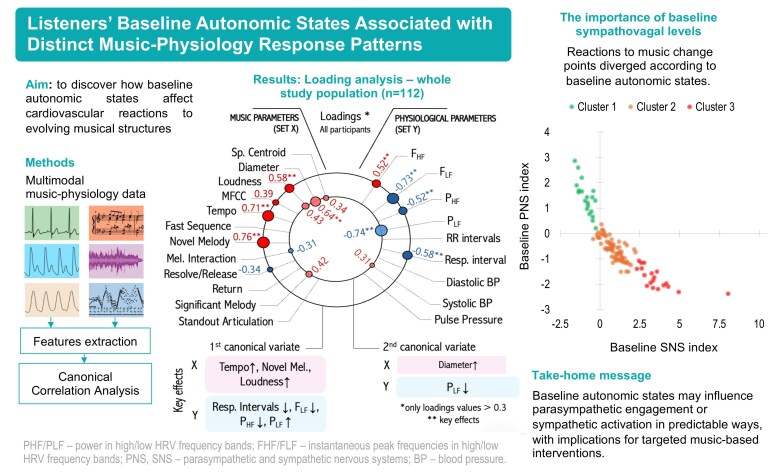

## Introduction

An essential aspect of music listening is the experience of changes in acoustic properties, often described as musical prosody or expressivity. Variations in tempo, loudness, or articulation shape listeners’ perceptual experience and are central to explaining physiological responses to music. As Debussy remarked, ‘music is the space between the notes’: beyond sectional form, it is the expressive shaping of musical structure that defines listening. Such expressive dynamics may also underpin how music influences the autonomic nervous system (ANS), with implications for cardiovascular health.

Music has been explored as a non-invasive tool for stress reduction, rehabilitation, and cardiovascular therapy. Although evidence suggests that music can modulate ANS activity and support cardiovascular disease (CVD) management, clinical findings remain inconsistent. Many studies rely on whole-piece comparisons to silence or other pieces,^1–6^ sometimes leading to overgeneralized conclusions like ‘classical music is relaxing’.^4,7^ These approaches obscure fine-grained, event-based physiological reactions and limit the potential for personalized music-based interventions.

Recent advances in music information retrieval (MIR) enable segmentation of musical signals into change points—transitions reflecting shifts in the distributional properties of acoustic features.^8^ Change point detection has been applied to tonal context,^9^ dynamics,^10^ and MIDI features such as pitch and velocity,^11^ and listeners can also annotate structural boundaries in music.^12^ Beyond music, change point methods are widely used across disciplines, including physiology, where they have been applied to skin conductance, RR intervals, and movement data^13–19^ Studies linking musical boundaries to physiological responses indicate that crescendos, tension–resolution patterns, and melodic transitions predict changes in respiration, heart rate, and skin conductance^18,20–22^ However, a generalizable framework integrating computational and physiological change points in the context of cardiovascular responses is still lacking.

To address this gap, we developed an event-based framework linking expressive musical changes to physiological dynamics using canonical correlation analysis (CCA). CCA, widely used in biomedical research to relate multivariate datasets such as brain networks and clinical symptoms,^23^ identifies shared structure between two sets of variables. Here, we adapt this approach to music–physiology data and introduce change point connectivity graphs to visualize how musical and physiological features jointly contribute to listeners’ responses.

Crucially, we examined these associations in the context of baseline autonomic balance. Baseline autonomic activity shapes stress reactivity^24,25^ and may similarly influence responses to expressive musical changes, yet this has been little studied. Engagement with non-tranquilizing music can act as a mild stressor, increasing heart rate and blood pressure,^4,26,27^ with responses differing between individuals. We therefore stratified participants by parasympathetic–sympathetic balance to test whether baseline autonomic state modulates music–physiology coupling and further explored response phenotypes using cluster-based analyses.

Overall, this study integrates computational change point detection, expert musical annotation, and multivariate analysis to characterize fine-grained music–physiology interactions. Understanding these dynamics is a key step towards developing personalized music-based interventions that promote relaxation or controlled arousal, advancing the field of music theranostics: music-based digital therapeutics and precision diagnostics.

## Methods

### Data collection

**Figure 1:**
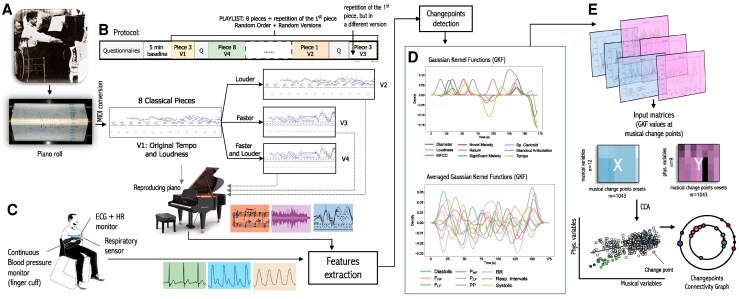
Data preparation for CCA. (*A*) Eight music pieces performed by famous pianists (including Claude Debussy, shown in the figure), reconstructed as MIDI files. Versions (V2-V4) of the original (V1) with altered loudness and/or tempo were prepared and rendered on a reproducing piano. The MIDI files, music scores, and audio signals from the rendered performances were sources of the musical features; (*B*) The study protocol included a questionnaire on demographic information and music sophistication, a 5-minute baseline, and the sequence of music pieces and versions played in random order; (*C*) Data collection from listeners employed three monitoring devices: ECG + HR chest belt, respiratory sensor, and continuous BP monitor (finger cuff). The signals from these sensors were used for the extraction of physiological variables; (*D*) Each change point for a given variable (musical and physiological) generates a Gaussian kernel function, the sum of which forms a time series; (*E*) The values at the musical change point onsets, taken from Gaussian time series from musical variables and averaged times series (over participants who listened to a given piece), create input matrices for calculating canonical variates and creating change points connectivity graphs.

The methodology and data processing for CCA is shown in *Figure 1*. Data were drawn from the HeartFM study, in which participants listened to nine expressive performances of eight Western classical piano pieces rendered on a reproducing piano. Each piece was presented in its original or altered form (e.g. louder, faster, or louder + faster), with the order and versions randomly selected; the first and last performances were different versions of the same piece. Altered versions were designed to isolate the effects of tempo and/or loudness while preserving other musical features. The repertoire was selected to span a wide range of musical structures and expressive styles and was sourced from Bösendorfer’s Legendary Artist Performance database (as MIDI files), including works by Sergei Prokofiev and Claude Debussy (it was possible by restoring their performances from the piano rolls.^28^) Details of the pieces, performers, and versions are provided in Supplementary data online, *Table S1* (see Supplementary Materials).

Prior to data collection, participants provided written informed consent, completed a questionnaire assessing musical sophistication (reduced Gold-MSI,^29^) and supplied demographic information. After a 5-min seated baseline recording, participants listened to the music rendered through the reproducing piano at a distance of approximately 3 m. Short silent intervals separated successive pieces, during which participants briefly shared impressions. Post-session feedback was collected but not analysed quantitatively; it served to monitor attention and engagement.

Inclusion criteria required participants to be ≥18 years old, have no hearing impairment, and be able to provide informed consent. The study conformed to the Declaration of Helsinki and was approved by the Oxford C Research Ethics Committee (IRAS 242471) and the Research Ethics Office at King’s College London (MRPP-22/23-34904).

### Physiological data

ECG signal and RR intervals were acquired by a Polar H10 (Polar Electro Oy, Kempele, Finland) sensor centred on the sternum using a textile electrode strap. Respiratory signals were registered using a BIOPAC (BIOPAC Systems, Goleta, USA) respiration belt placed slightly below the ECG strap. The ECG and respiration data were collected concurrently and visualized using the HeartFM mobile and desktop visualization apps.^30^ Continuous blood pressure (BP) waveforms were collected using the finger cuffs of the CNAP monitor (CNSystems, Graz, Austria). A systolic (SYS), diastolic (DIA) BP, and pulse pressure (PP = SYS—DIA) were automatically extracted from each pulse wave.

### Music data

All music rendered was recorded on a Zoom H5 (Zoom, Tokyo, JP) handheld recorder placed in the same position as the listeners’ chair. The recordings were aligned with the MIDI signals and used for calculating music-related signals: loudness (in sones), tempo (based on the onsets of beats), harmonic tension diameter, which is the longest distance between the pitch positions in the spiral array space at every beat,^31–33^ spectral centroid (the ‘balance point’ of the spectrum, a centroid of the frequencies in the music,^34^) and mel-frequency cepstral coefficients (MFCC, a measure of timbre.^35^)

### Data processing

All collected signals were resampled to 5 Hz. RR intervals were manually inspected for ectopic beats, which were removed, and subsequently filtered using a high-pass 4th order Butterworth filter with a cut-off frequency of 0.03 Hz before calculating the HRV spectral parameters, such as instantaneous peak frequencies $F_{LF}$, $F_{HF}$ and the instantaneous powers $P_{LF}$, $P_{HF}$ of low (LF) and (high) HF frequency bands in a spectrogram obtained using the short-time Fourier transform [STFT using 50-sample Hamming windows and Python *stft*() function with default configuration from the *scipy.signal* library]. Power in the HF band corresponds to heart rate (or RR interval) oscillations predominantly driven by parasympathetic (vagal) activity, particularly through respiratory sinus arrhythmia (RSA). The $P_{HF}$ quantifies the variance (energy) of RR interval fluctuations, while the $F_{HF}$ indicates the dominant oscillatory frequency in that band. The LF band captures slower RR interval oscillations, influenced by both sympathetic and parasympathetic input, but primarily reflecting baroreflex-mediated autonomic regulation. An increase/decrease in the power spectra ($P_{HF}$ and $P_{LF}$) expresses the shifts in the autonomic activity in time. The peak frequency values ($F_{HF}$ and $F_{LF}$) are linked with the respiration patterns and they should be interpreted together with the respiratory interval series. For example, an increase in $F_{HF}$ with a decrease in respiratory intervals is expected and reflects faster respiration, shifting the RSA to a higher frequency. In turn, a decrease in $F_{LF}$ might reflect a shift in RSA from the LF to the HF band due to a musical change point.^36^ While the LF component was defined by a standard range of frequencies (0.04–0.15 Hz), the range for the HF component was made time-varying (instead of a fixed standard range of 0.15–0.4 Hz) and dependent on the respiratory frequency $F_{r}(t)$ (based on the respiratory intervals extracted from the respiratory signal), $F_{r}(t)\pm [-0.125;0.125]$ Hz. This methodology was widely described in,^37^ where it was found to improve the estimation of RSA in continuous time. Examples of the spectrograms are shown in the Supplementary Materials (Supplementary data online, *Figure S5*).

The respiratory intervals (resp. intervals) were detected automatically from detrended and filtered signals measured by the respiration belt using a low-pass 4th order Butterworth filter with 1 Hz cut-off frequency; all intervals were checked and corrected manually.

The baseline RR intervals were also used to calculate the balance between the parasympathetic (PNS) and sympathetic (SNS) nervous systems prior to listening to music. PNS and SNS indexes were calculated from the RR interval series collected during baseline in the Kubios HRV Software.^38^ Each index uses three standardized HRV parameters associated with PNS (mean RR interval, RMSSD, and Poincaré Plot Index SD1 in Normalized Units) and SNS (mean HR, Baevsky’s Stress Index, and Poincaré Plot Index SD2 in Normalized Units) branches. The index values were used to cluster the participants into three groups (using k-means clustering), reflecting the three principal variants of baseline autonomic control: parasympathetic-dominant (high PNS and low SNS), balanced (comparable PNS and SNS), and sympathetic-dominant profiles (low PNS and high SNS indexes).

### Change points and interpretation maps

#### Automatically detected change points

The automatic detection of the change points is carried out on continuous musical and physiological signals collected during the study.

We applied a unified change point detection framework across musical and physiological signals. For each signal, candidate change points were identified using two established algorithms: *cpt.mean()*^39^ for normally distributed inputs and *cpt.np()*^40^ for non-parametric distributions. To ensure robustness against algorithmic and parameter choices, change point detection was repeated across a broad set of parameter combinations, including penalty values, segmentation methods, normalization options, and sampling resolutions.

Consistency was defined operationally as the repeated detection of a change point at the same temporal location across multiple parameter configurations. Specifically, we constructed a probabilistic representation of change point occurrence over time and retained only those events whose detection frequency exceeded a predefined threshold, indicating stable support across parameterizations. This ensemble-based approach reduces sensitivity to any single configuration and favours reproducible structural transitions.

To prevent spurious clustering of closely spaced detections, a minimum spacing threshold was applied: change points occurring within a short temporal window were merged and represented by a single event. This constraint reflects the limited temporal resolution at which meaningful physiological and musical transitions can be distinguished and avoids over-segmentation of gradual changes. A schematic overview of the framework and validation examples are provided in the Supplementary Materials (Supplementary data online, *Figures S11–S13*).

#### Interpretation map (manually detected change points)

Two experts jointly annotated the onsets of compound musical structures while listening to the music under the same conditions as the participants. These annotations reflect expert interpretations of expressive and prosodic musical structures and constitute a novel set of categories, referred to as the *Interpretation Map*. It captures performed expressive elements likely to influence listeners’ physiological responses and has previously been applied to model musicians’ RR intervals using linear mixed models.^41^

In the present study, annotated events were grouped into 16 categories, including Novel Melody, Return, Build-up/Significant Crescendo, Climax, Resolve/Release, Melodic Interaction, Significant Melody, Significant Silence, Standout Articulation, Emphasis, Runs/Fast Sequence, Swell, Tension, Instability, Drop/Gentle, and Arrival (definitions of these categories were described in the Supplementary data online, *Table S2*). The onsets of these annotated events were combined with automatically detected musical change points and are collectively referred to as musical change points.

Musical and physiological change points were then transformed into time series using Gaussian kernel functions (GKF) centred at their onsets, separately for each piece and signal. The resulting GKF-based time series served as input to the canonical correlation analysis. A detailed description of the annotation-to-GKF procedure is provided in the Supplementary Materials.

### Statistical analysis

#### Canonical correlation analysis

Canonical correlation analysis (CCA) is a multivariate statistical method used to quantify the relationship between two sets of variables. In this study, CCA was used to examine associations between musical structure and physiological dynamics, with musical change points serving as the fundamental observational units. Accordingly, each row of the input matrices corresponds to a single musical change point, defined as a consistent change detected in the musical feature time series.

Let $X_{n\times p}$ denote the musical input matrix and $Y_{n\times q}$, the physiological input matrix, where n is the number of musical change points and *p* and *q* are the numbers of musical (*P* = 12) and physiological (*q* = 9) variables, respectively. The matrices were constructed such that rows of X and Y are temporally aligned, representing paired musical–physiological events at each change point.

CCA identifies pairs of linear combinations of the variables in X and Y that are maximally correlated. Specifically, CCA estimates weight vectors u and v such that the correlation between the canonical variates $U=uX^{T}$ and $V=vY^{T}$:


$$\begin{array}{r} \mathrm{maximize}\;uX^{T}Y^{T}v,\\ \mathrm{subject}\;\mathrm{to}\;u^{T}X^{T}\mathrm{Xu}\leq 1,\;v^{T}X^{T}\mathrm{Xv}\leq 1 \end{array}$$

The first pair of canonical variates (U₁, V₁) captures the strongest shared association between the two datasets. Subsequent pairs (Uᵢ, Vᵢ) are derived under orthogonality constraints and describe progressively weaker, independent modes of covariation. In addition to the canonical correlation coefficients, relative covariance measures were computed to estimate the proportion of variability explained by each canonical mode.

#### Construction of input matrices

The musical input matrix X was not derived directly from continuous feature time series. Instead, change points detected in musical features were first converted into continuous representations by convolving each change point onset with a Gaussian kernel (GK). The values of these GK time series were then sampled at the onset of each musical change point to populate the rows of X.

The physiological input matrix Y was constructed analogously. Change points detected in physiological signals were transformed into GK time series, and values were sampled at the same musical change-point onsets. This procedure ensured that each row of X and Y represents a temporally aligned musical–physiological interaction defined at the level of change points rather than raw signal amplitudes. A detailed description of the change point detection and GK transformation procedures is provided in the Supplementary Materials. Because multiple participants listened to the same musical pieces, physiological responses were averaged across all listeners exposed to a given piece prior to CCA. This design choice enabled a unified multivariate analysis across the complete stimulus set while preserving alignment between musical structure and physiological dynamics. Consequently, the resulting CCA captures aggregated associations between musical and physiological variables at the piece level rather than individual-level predictive relationships.

#### Canonical loadings

Apart from the canonical weights, within-set correlations between each canonical variate and the original variables in **X** and **Y** were calculated; these are referred to as canonical loadings (structural coefficients), which are generally more interpretable than the weights.^42^ High absolute loading values indicate variables that contribute strongly to a given canonical variate. Interpretation focused on pairs of canonical variates with the highest and statistically significant canonical correlations, linking variables with the largest absolute loadings in $U_{i}$ and $V_{i}$. The relative signs of the loadings indicate the direction of association between variables in the two datasets: opposite signs imply a negative association, whereas the same sign indicates a positive association.

A special case involves loadings associated with manually annotated musical change points (e.g. Novel Melody). Unlike continuous musical features such as loudness, these annotations represent compound structural events that cannot be described by a simple directional change. Their interpretation, therefore, relies on the sign of the associated physiological loading. For example, a negative loading for Novel Melody combined with a positive loading for respiratory interval indicates a decrease in respiratory intervals following the occurrence of a novel melody. In contrast, loadings of the same sign indicate an increase.

Statistical significance and selection of canonical variates were assessed using Wilks’ Lambda, surrogate-based testing, and permutation testing (see Supplementary Materials), and exploratory subject-wise CCA analyses were additionally performed as robustness and heterogeneity checks, without replacing the primary group-level multivariate analysis.

#### Change points connectivity graphs

We developed a new way to visualize the key effects observed in CCA, called change point connectivity graphs. These graphs display loading values from selected canonical variates as concentric black circles, with the outermost circle corresponding to the first variate. Individual loadings are shown as smaller red (positive) or blue (negative) circles, with musical variables on the left and physiological variables on the right. For clarity, only loadings with absolute values greater than 0.3 are shown. Arrows indicate the direction of association between musical and physiological variables based on the signs of their loadings, and the dominant effects for each variate are summarized below each graph.

#### Effect of age, gender, personal musical characteristics on music-physiology patterns

To examine whether individual characteristics modulate the multivariate music–physiology coupling identified at the group level, we analysed how individual response patterns relate to baseline physiological and demographic features. Specifically, CCA was performed separately for each participant (i.e. the size of the input matrices was determined by the number of musical change points in the pieces comprising a given listener’s playlist), yielding individual sets of canonical loadings.

Each individual loading set was compared with the loadings obtained for the entire study group using a cosine similarity measure, quantifying the degree of directional alignment between individual and group-level multivariate patterns. These similarity values were then analysed using generalized additive models (GAMs) as the dependent variable, with baseline physiological indices (SNS/PNS indices, systolic and diastolic blood pressure) and demographic characteristics (age, gender, musicianship level, and music preference) included as predictors.

GAMs have previously been applied in neuroimaging studies, such as by Xia et al.^43^ to examine age- and sex-related effects on multivariate patterns derived via CCA. Detailed model specification, and full statistical results are provided in the Supplementary Materials.

#### Differences analysis at musical change points

As an additional quantitative analysis, we calculated the differences in the mean values of physiological signals across 10-second windows before and after each musical change point. This approach provides a direct observation and quantitative insights into the instantaneous response of the listener to musical change points. The stability of the results was validated for 8-s and 12-s windows and presented in Supplementary Materials (see Supplementary data online, *Figure S4*).

The differences are reported as the mean value and 95% CI in the signals’ units. We used a paired *t*-test or Wilcoxon test (depending on the normality of the distribution of differences) to verify whether the differences between the windows before and after the change point are significant. We analysed each musical category from the Interpretation Map and the change points from acoustic signals separately, providing an increase (denoted as ↑) or decrease (↓) in the musical signal (17 categories in total). To reduce the risk of Type I errors, the Bonferroni correction was used to analyse each of the nine physiological signals.

## Results

### Participants

Data were obtained from 112 participants (63 females and 49 males) with a mean age of 43.9±16.0 years (range 21–79 years). Based on the PNS and SNS normalized indexes calculated from baseline RR intervals, three clusters were created using the K-means clustering: cluster 1—with high parasympathetic index and low sympathetic index (*n* = 20), cluster 2—sympathovagal balance (*n* = 73), cluster 3—high sympathetic index and low parasympathetic index (*n* = 19; PNS and SNS indexes grouped in clusters were shown in *Figure 3B*). The demographic profiles between these clusters were compared in Supplementary data online, *Figure S1* in the Supplementary Materials. Twenty-five participants (22%) had elevated baseline blood pressure at the baseline (systolic >140 mmHg or diastolic >90 mmHg). Twenty-three participants (21%) were assessed with a high level of musicianship.

Based on the questionnaire about music sophistication and experience, 55 participants (49%) had at least one year of formal musical training (14 participants for 0.5–1 years, 21 for 2–3 years, 11 for 4–6 years, and 12 for more than 7 years), 78 participants (70.0%) declared a regular, daily practice of a musical instrument (including voice) for at least one year (26 participants for 1–3 years, 27 participants for 3–9 years, and 25 participants for more than 10 years). Moreover, classical music was a favourite genre for 37 (33%) participants.

### Change points annotations

The number of detected change points in all music pieces was 813: 520 were detected automatically in tempo (156), loudness (152), spectral centroid (79), MFCC (39), diameter signals (94), and the experts annotated manually 293 onsets (Interpretation Map): 71 of Significant Melody, 44 of Novel Melody, 32 of Return, 25 of Melodic Interaction, 25 of Standout Articulation, 19 of Run/Fast Sequence, and 19 of Resolve/Release. The following under-represented categories (<15 annotations) were not considered in the analysis: Build-up (13 annotations), Climax (11), Emphasis (10), Swell (6), Significant Silence (4), Tension (4), Instability (4), Arrival (3), Drop/Gentle (2), Fast Sequence Release (1). The number of change points in each piece is shown in Supplementary data online, *Table S2* of the Supplementary Materials.

### Aggregate patterns: canonical correlations for the whole study group

The first two canonical variates were selected for the final analysis using the multistep procedure described in the Supplementary Materials to ensure the robustness of the effects represented by the loading values. For the whole study group, canonical correlations for these variates equaled 0.56 and 0.36, respectively, both p<0.001, Wilks’ lambda. They identified two distinct physiological response domains: respiratory dynamics (first variate) and cardiac control, as measured by RR intervals (second variate).

In the first variate, Novel Melody and Tempo showed the strongest associations, linked with shortened respiratory intervals and shifts in spectral power (decrease in $F_{LF}$, increase in $F_{HF}$). These patterns reflect faster, shallower breathing and parasympathetic withdrawal in response to musical novelty and increases in intensity. In other words, expressive changes acted as acute arousal triggers.

The second variate was characterized by autonomic modulation of cardiac intervals, with high loadings for $P_{LF}$ and vascular diameter. This suggests that beyond respiratory coupling, music also engages cardiovascular control systems, particularly through sympathetic influences on vascular tone and heart rhythm. In summary, we observe two primary pathways: respiratory entrainment and autonomic modulation of cardiac control. Rather than uniform ‘relaxation effects,’ music evokes specific, event-based adjustments that depend on the underlying musical features.

#### Differences in physiological signals before and after musical change points

When aggregated across the whole study group, several musical change-point categories showed significant physiological changes after Bonferroni correction, including Novel Melody, Spectral Centroid↑, Diameter↑/↓, Loudness↑, MFCC↑, and Tempo↑ (*Figure 3*; full numerical results are presented in Supplementary data online, *Table S3* in the Supplementary Materials). Overall, these events produced response patterns consistent with sympathetic activation, often accompanied by changes in respiration.

Novel Melody elicited the strongest respiratory effects, with shortened respiratory intervals ($p=2\cdot 10^{-8}$), reduced $F_{LF}$ ($p=3\cdot 10^{-10}$), and increased $F_{HF}$ ($p=3.4\cdot 10^{-4}$). Loudness↑ and Tempo↑ had similar effects, decreasing respiratory and RR intervals while shifting HRV towards higher-frequency components, indicative of acute autonomic adjustment.

In contrast, MFCC↑ events were linked with broader cardiovascular changes, including increased diastolic blood pressure ($p=1\cdot 10^{-7}$) and elevated $F_{HF}$ ($p=4\cdot 10^{-8}$). Diameter changes showed vascular-specific responses: Diameter↑ corresponded with shorter RR intervals, whereas Diameter↓ was associated with reduced pulse pressure.

These direct before–and–after comparisons reinforce the CCA-derived patterns, where Novel Melody, Tempo, and Loudness consistently loaded with respiratory and RR interval changes, while MFCC and vascular features contributed more strongly to blood pressure and pulse dynamics.

### Subgroup patterns: canonical correlations in PNS-SNS clusters

In the exploratory within-cluster CCA, baseline autonomic balance was strongly linked to distinct patterns of response to musical structures (*Figure 4*). Listeners with high parasympathetic tone at baseline (Cluster 1, n=20;18%) showed responses consistent with further parasympathetic activation (in the first variate): Novel Melody, Tempo, and spectral changes were associated with lengthened RR intervals and altered respiration intervals, reflecting relaxation-oriented dynamics. This suggests that expressive novelty in music can reinforce parasympathetic regulation in listeners already predisposed to relaxation.

By contrast, listeners with high sympathetic tone (Cluster 3, n=19;17%) displayed the opposite profile. Increases in Loudness, Tempo, and related acoustic features were associated with shortened RR intervals and reduced $P_{HF}$ (in the first variate), reflecting sympathetic activation. Importantly, Novel Melody was not a strong driver in this group, suggesting that intensity rather than structural novelty governs their responses. For such individuals, music may act as a mild stressor or controlled arousal stimulus, which could be either beneficial (e.g. for motivation) or potentially adverse in vulnerable patients.

The balanced group (Cluster 2), comprising the majority of participants (n=73;65%), displayed intermediate patterns that resembled the overall cohort: Novel Melody and Tempo had a strong influence on responses, with respiratory changes as the dominant physiological correlate. Note that the number of musical change points analyzed was comparable across clusters and to that of the full cohort; however, cluster-specific analyses differed in the number of participants contributing to the averaged physiological responses at each change point.

### Individual patterns: subject-wise CCA and the effect of inter-subject differences on music-physiology patterns

**Figure 2:**
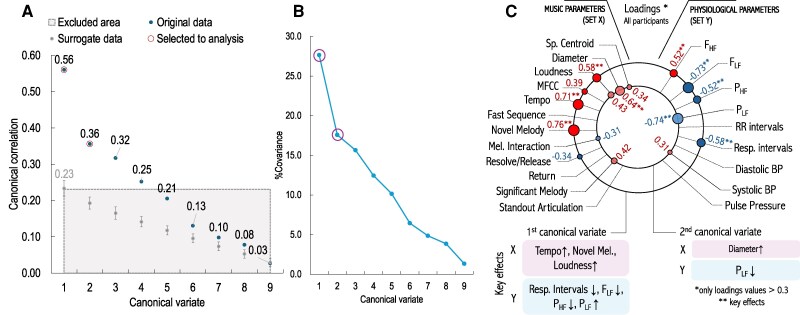
Results of CCA on the whole study group; (*A*) Canonical correlation coefficients for all variates for original and surrogate data. The first two variates selected for analysis were marked with a purple circle. The grey area includes variates that were lower than the maximum mean correlation coefficient found in the surrogate data; (*B*) Percentage covariance for each canonical variate; (*C*) Change point connectivity graph. The size and colour of the circles represent the value and sign of the loadings, respectively (red: positive value, blue: negative value). Only values > 0.3 were displayed for clarity. The key effects of each variable are listed below the plot.

The subject-wise exploratory CCA demonstrated substantial inter-individual heterogeneity in magnitude while showing non-random directional tendencies for several key couplings observed in the primary analysis (*Figure 2*), supporting the aggregated-level interpretation while explicitly acknowledging individual variability. The directional alignments were presented in Supplementary data online, *Table S7* in the Supplementary Materials.

**Figure 3:**
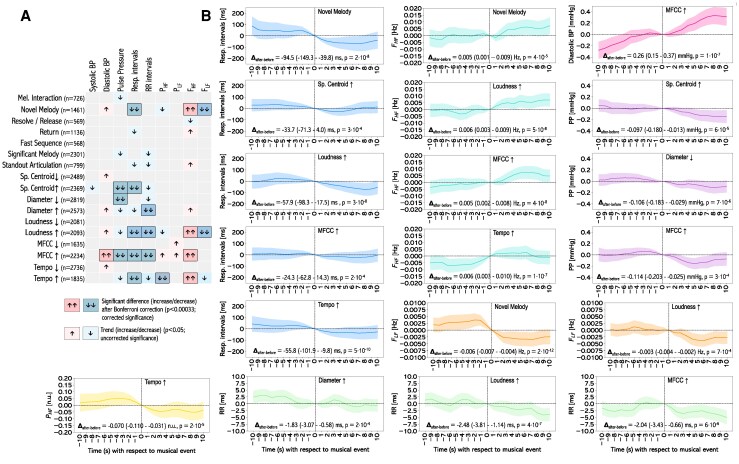
The results of the analysis of the difference in physiological signals before and after musical change point; (*A*) A diagram depicting the direction of changes in physiological signals for each musical category. Two arrows mean that the difference was significant (after using Bonferroni correction, considering all 153 combinations of features, p<0.00033), while a single arrow indicates a trend (p<0.05); (*B*) Mean values of physiological signals before and after musical onsets, where a significant difference was found (two arrows in diagram *A*).

**Figure 4:**
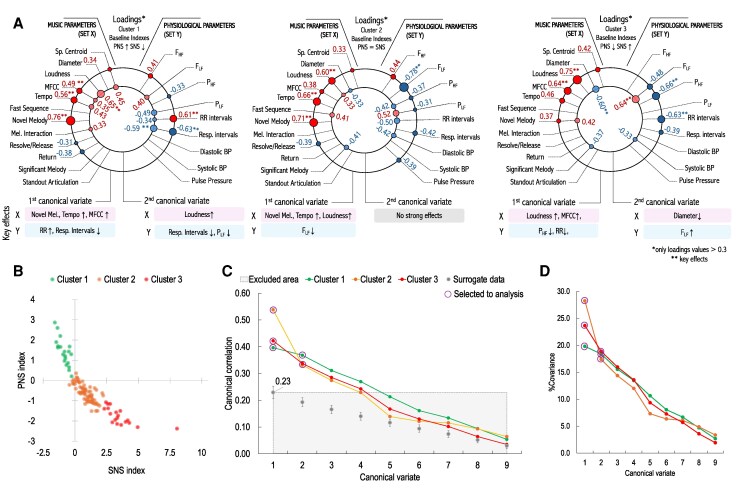
Results of CCA in three clusters based on the baseline PNS and SNS indexes; (*A*) Change point connectivity graphs. The size and colour of the circles represent the value and sign of the loadings, respectively (red-positive value, blue-negative value). Only values > 0.3 were displayed for clarity. The main effects of each variable are listed below the plot; (*B*) A scatter plot of the PNS and SNS indexes calculated for the baseline RR intervals for each participant. The clusters were created using the k-means method; (*C*) Canonical correlation coefficients for all variates for original and surrogate data. The variates selected for analysis were marked with a circle. The excluded area includes variates that were lower than the maximum mean correlation coefficient found in the surrogate data; (*D*) Percentage covariance for each canonical variate.

**Figure 5:**
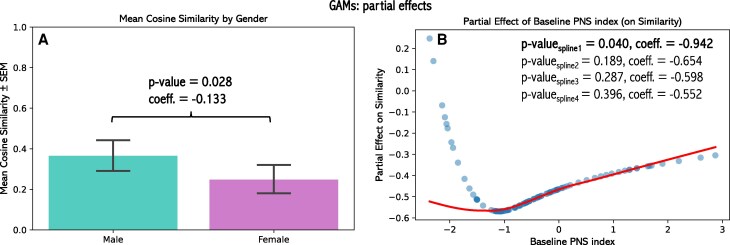
Partial effects from generalized additive models (GAMs) illustrating the influence of gender (*A*) and baseline parasympathetic nervous system (PNS index; *B*) on the similarity between individual and group-level canonical loadings. Both variables were identified as significant predictors of similarity in the model.

In the GAMs analysis, two variables showed nominally significant effects: baseline PNS index (*P* = 0.040, $b_{\mathrm{splin}e_{1}}$ = −0.942) and gender (*P* = 0.028, coefficient = −0.1332), with partial effects illustrated in *Figure 5* and full results reported in the Supplementary Materials (Supplementary data online, *Table S5*, Supplementary data online, *Figure S10*).

Higher baseline PNS index was associated with lower cosine similarity to the group-level loading pattern, indicating greater individual deviation from the canonical profile. Female participants also exhibited lower similarity to the group-level pattern than males, suggesting more distinct physiological response profiles despite their larger representation in the cohort. However, neither effect remained significant after correction for multiple comparisons (Benjamini–Hochberg) and should therefore be interpreted as indicative trends rather than definitive findings.

Notably, the PNS-related trend is consistent with subgroup-specific CCA results, in which participants with high baseline PNS levels exhibited distinct associations between musical variables and RR interval change points, including RR interval increases during rising intensity and the emergence of novel melodies (*Figure 4A*).

## Discussion

Our findings suggest that individuals’ baseline autonomic balance influence whether expressive musical changes promote parasympathetic relaxation or sympathetic arousal, providing a physiological basis for personalizing music-based cardiovascular interventions. Rather than producing a uniform ‘relaxing’ effect, music operates through discrete events—such as novel melodies, tempo shifts, and intensity changes—that function as micro-interventions shaping respiration and cardiac dynamics.

Across the cohort, novelty emerged as the most consistent driver of physiological change, typically shortening respiratory intervals and shifting spectral power towards increased arousal. These results extend earlier observations that crescendos and tension–resolution patterns synchronize with respiration and heart rate,^20,21^ demonstrating that autonomic engagement is driven not only by overall musical profile but also by the occurrence of novel events. Notably, most significant physiological differences were associated with increases in continuous musical features (e.g. loudness, tempo, spectral complexity), rather than decreases. This pattern suggests an overall engaging and arousing character of the expressive music stimuli and provides an important physiological context for interpreting the multivariate associations identified by CCA.

Importantly, responses diverged according to baseline physiology. Listeners with high parasympathetic tone showed lengthened RR intervals and parasympathetic reinforcement in response to novel melodies and tempo changes, whereas those with sympathetic dominance exhibited shortened RR intervals and reduced $P_{HF}$ during increases in loudness and in spectral features. The balanced group showed intermediate responses. Together, these findings indicate that the direction and magnitude of music-induced autonomic modulation depend on the listener’s baseline state, which may be a marker of their autonomic tendency.

This baseline dependence has potential clinical implications. For parasympathetic-dominant individuals, music rich in novelty may further promote relaxation, whereas for sympathetic-dominant individuals similar stimuli may elicit arousal, which could be beneficial in motivational or rehabilitative contexts but less desirable in vulnerable patients. In both cases, musical change points act as targeted stimuli that modulate autonomic state, highlighting their potential as building blocks for personalized therapeutic playlists.

The general arousal observed across the cohort is consistent with eustress—a form of positive stress. Expressive musical events can elicit stress-like physiological patterns while being experienced as positive, aligning with evidence that eustress reduces cortisol and salivary alpha-amylase.^44,45^ This distinction helps reconcile inconsistencies in the literature: while tranquillizing music can induce group-level relaxation,^7,46,47^ expressive classical music resists simple categorization,^3^ underscoring the context-dependent and individualized nature of music–physiology coupling.

Exploratory analyses using GAMs suggested possible gender-related differences and reinforced the role of baseline parasympathetic tone; however, these effects should be interpreted cautiously, as they did not survive correction for multiple comparisons.

This study has several limitations. CCA was performed on piece-level–averaged physiological responses and therefore captures group-level coupling rather than subject-level prediction. Although the number of observations entering CCA was comparable across clusters, cluster-specific analyses relied on responses averaged over fewer participants and are therefore exploratory. Extending this framework to subject-level modeling will require datasets with complete stimulus overlap or explicitly hierarchical designs. Interpretation Maps were based on expert annotations, which may vary from individual perception. The cohort comprised healthy British adults exposed to Western classical music; generalizability to other cultures, genres, and clinical populations (e.g. hypertension or autonomic dysfunction) warrants further study.

Despite these caveats, the study demonstrates that musical change points are powerful drivers of physiological responses and that baseline autonomic state may be an indicator of whether these responses promote relaxation or arousal. This baseline-informed, event-based framework moves beyond generic claims about music’s ‘relaxing’ effect and provides a path towards precision music therapeutics. By aligning musical features with individual autonomic profiles, it may be possible to design personalized, non-invasive interventions not only for cardiovascular prevention, rehabilitation, stress management but also for cardiovascular imaging and practice. For example, autonomic response phenotypes derived from music–physiology coupling could be used to standardize or monitor autonomic state prior to cardiovascular imaging, where sympathetic activation is known to affect heart rate, vascular tone, and image quality. In this context, music-based modulation could serve as a non-pharmacological tool to reduce autonomic variability before imaging acquisition. In addition, music–physiology response patterns could be integrated with imaging-derived biomarkers (e.g. ventricular function, vascular stiffness, or myocardial strain) in rehabilitation or risk stratification, providing complementary information on autonomic regulation. While these applications remain exploratory, they illustrate how change-point-based music–physiology profiling could support future precision approaches in cardiovascular diagnostics and rehabilitation.

## Conclusions

Our findings show that baseline autonomic state is an important factor associated with whether expressive musical changes evoke increased parasympathetic activity or sympathetic arousal. This principle provides a physiological foundation for tailoring music as a precision intervention in cardiovascular care. We demonstrate that music acts through discrete events—novel melodies, tempo shifts, changes in intensity—that function as micro-interventions modulating respiration and cardiac dynamics.

Future randomized clinical trials should test whether matching music interventions to patients’ baseline autonomic profiles improves outcomes in cardiovascular prevention, rehabilitation, and stress management. By integrating event-based analysis with baseline physiology, this study provides a step towards clinically actionable, non-pharmacological music therapeutics.

## Supplementary Material

qyag013_Supplementary_Data

## Data Availability

The datasets generated and/or analysed during the current study are not publicly available as they are part of a larger planned data publication but data and code (Python, R) used for the analysis are available from the corresponding author on reasonable request.

## Lead author biography

**Mateusz Soliński** is a specialist in multimodal biomedical data analysis and artificial intelligence in medicine, with extensive experience in medical-device development, data-science leadership, and academic research. His work examines how expressive musical parameters influence autonomic responses and how music perception interacts with physiological processes. He holds a PhD in Physical Sciences from the Faculty of Physics at Warsaw University of Technology (2022), where he worked within the Cardiovascular Physics Group. Alongside his academic career, including postdoctoral research at King’s College London, UK (2022–2025) and at Tampere University, Finland (2025-), he gained industry experience as a Data Scientist at medical companies and start-ups (Medicalgorithmics, AioCare), contributing to the development of medical devices and telemedicine systems. He has participated in numerous commercial projects, clinical trials, and research challenges involving electrophysiological signal analysis, particularly ECG, PPG, and heart-rate variability. He is also a co-founder of OpenPPG, a mobile research application for recording pulse waveforms using a smartphone camera.

## References

[1] Bernardi L, Porta C, Sleight P. Cardiovascular, cerebrovascular, and respiratory changes induced by different types of music in musicians and non-musicians: the importance of silence. Heart 2005;92:445–52. 16199412 10.1136/hrt.2005.064600 PMC1860846

[2] Loomba RS, Arora R, Shah PH, Chandrasekar S, Molnar J. Effects of music on systolic blood pressure, diastolic blood pressure, and heart rate: a meta-analysis. Indian Heart J 2012;64:309–13. 22664817 10.1016/S0019-4832(12)60094-7 PMC3860955

[3] Trappe H-J . Music and medicine: the effects of music on the human being. Appl Cardiopulm Pathophysiol 2012;16:133–42.

[4] Koelsch S, Jäncke L. Music and the heart. Eur Heart J 2015;36:3043–9. 26354957 10.1093/eurheartj/ehv430

[5] Mojtabavi H, Saghazadeh A, Valenti VE, Rezaei N. Can music influence cardiac autonomic system? A systematic review and narrative synthesis to evaluate its impact on heart rate variability. Complement Ther Clin Pract 2020;39:101162. 32379689 10.1016/j.ctcp.2020.101162

[6] Kulinski J, Ofori EK, Visotcky A, Smith A, Sparapani R, Fleg JL. Effects of music on the cardiovascular system. Trends Cardiovasc Med 2022;32:390–8. 34237410 10.1016/j.tcm.2021.06.004 PMC8727633

[7] Ellis RJ, Thayer JF. Music and autonomic nervous system (dys)function. Music Percept 2010;27:317–26. 21197136 10.1525/mp.2010.27.4.317 PMC3011183

[8] Killick R, Fearnhead P, Eckley IA. Optimal detection of changepoints with a linear computational cost. J Am Stat Assoc 2012;107:1590–8.

[9] Chew E . Regards on two regards by Messiaen: post-tonal music segmentation using pitch context distances in the spiral array. J New Music Res 2005;34:341–54.

[10] Kosta K, Bandtlow OF, Chew E. A change-point approach towards representing musical dynamics. In: Mathematics and Computation in Music. Cham, Switzerland: Springer International Publishing; 2015. p.179–84.

[11] Dean RT, Bailes F, Drummond J. Generative structures in improvisation: computational segmentation of keyboard performances. J New Music Res 2014;43:224–36.

[12] Chew E . COSMOS: computational shaping and modeling of musical structures. Front Psychol 2022;13:527539. 35712186 10.3389/fpsyg.2022.527539 PMC9197258

[13] Reeves J, Chen J, Wang XL, Lund R, Lu QQ. A review and comparison of changepoint detection techniques for climate data. J Appl Meteorol Climatol 2007;46:900–15.

[14] Jessa S, Mohammadnia A, Harutyunyan AS, Hulswit M, Varadharajan S, Lakkis H et al K27m in canonical and noncanonical H3 variants occurs in distinct oligodendroglial cell lineages in brain midline gliomas. Nat Genet 2022;54:1865–80. 36471070 10.1038/s41588-022-01205-w PMC9742294

[15] Rathcke T, Lin C-Y, Falk S, Bella SD. Tapping into linguistic rhythm. Lab Phonol 2021;12:11.

[16] Nam CFH, Aston JAD, Johansen AM. Quantifying the uncertainty in change points. J Time Ser Anal 2012;33:807–23.

[17] Rosenfield D, Zhou E, Wilhelm FH, Conrad A, Roth WT, Meuret AE. Change point analysis for longitudinal physiological data: detection of cardio-respiratory changes preceding panic attacks. Biol Psychol 2010;84:112–20. 20144682 10.1016/j.biopsycho.2010.01.020 PMC2978268

[18] Dean RT, Bailes F. Relationships between generated musical structure, performers’ physiological arousal and listener perceptions in solo piano improvisation. J New Music Res 2016;45:361–74.

[19] Dubatovka A, Mikhailova E, Zotov M, Novikov B. Algorithms for extracting mental activity phases from heart beat rate streams. In: *International Baltic Conference on Databases and Information Systems*. Riga, Latvia: Springer International Publishing; 2016. p113–25.

[20] Bernardi L, Porta C, Casucci G, Balsamo R, Bernardi NF, Fogari R et al Dynamic interactions between musical, cardiovascular, and cerebral rhythms in humans. Circulation 2009;119:3171–80. 19569263 10.1161/circulationaha.108.806174

[21] Tsai C-G, Chen C-P. Musical tension over time: listeners’ physiological responses to the ‘retransition’ in classical sonata form. J New Music Res 2015;44:271–86.

[22] Greer T, Ma B, Sachs M, Habibi A, Narayanan S. A multimodal view into music’s effect on human neural, physiological, and emotional experience. In: *Proceedings of the 27th ACM International Conference on Multimedia*. Nice, France: ACM; 2019.

[23] Wang H-T, Smallwood J, Mourao-Miranda J, Xia CH, Satterthwaite TD, Bassett DS et al Finding the needle in a high-dimensional haystack: canonical correlation analysis for neuroscientists. NeuroImage 2020;216:116745. 32278095 10.1016/j.neuroimage.2020.116745

[24] Kudielka BM, Schommer NC, Hellhammer DH, Kirschbaum C. Acute HPA axis responses, heart rate, and mood changes to psychosocial stress (TSST) in humans at different times of day. Psychoneuroendocrinology 2004;29:983–92. 15219648 10.1016/j.psyneuen.2003.08.009

[25] Hoareau V, Godin C, Dutheil F, Trousselard M. The effect of stress management programs on physiological and psychological components of stress: the influence of baseline physiological state. Appl Psychophysiol Biofeedback 2021;46:243–50. 33978903 10.1007/s10484-021-09508-0 PMC8325665

[26] Pope V, Soliński M, Lambiase P, Chew E. Raised blood pressure alters reactivity to musical features. In: *Congress of the European Society of Cardiology*, London, UK. 2024.

[27] Pope VC, Soliński M, Lambiase PD, Chew E. High blood pressure inhibits cardiovascular responsiveness to expressive classical music. Sci Rep 2025;15:10908. 40157971 10.1038/s41598-025-94341-2 PMC11954918

[28] Colmenares G, Escalante R, Sans JF, Surós R. Computational modeling of reproducing-piano rolls. Comput Music J 2011;35:58–75.

[29] Müllensiefen D, Gingras B, Musil J, Stewart L. The musicality of non-musicians: an index for assessing musical sophistication in the general population. PLoS One 2014;9:e89642. 24586929 10.1371/journal.pone.0089642 PMC3935919

[30] Chew E, Fyfe L, Picasso C, Lambiase P. Seeing music’s effect on the heart. Eur Heart J 2024;45:4359–63. 39405174 10.1093/eurheartj/ehae436

[31] Chew E . Towards a mathematical model of tonality. *Doctoral Dissertation*. Massachusetts Institute of Technology, 2000.

[32] Chew E . Mathematical and Computational Modeling of Tonality: Theory and Applications. New York, USA: Springer; 2013.

[33] Herremans D, Chew E. Tension ribbons: quantifying and visualising tonal tension. In: *Proceedings of the Second International Conference on Technologies for Music Notation and Representation (TENOR)*, p8–18, Cambridge, UK. 2016.

[34] Grey JM, Gordon JW. Perceptual effects of spectral modifications on musical timbres. J Acoust Soc Am 1978;63:1493–500.

[35] Mauch M, MacCallum RM, Levy M, Leroi AM. The evolution of popular music: USA 1960–2010. R Soc Open Sci 2015;2:150081. 26064663 10.1098/rsos.150081 PMC4453253

[36] Heart rate variability: standards of measurement, physiological interpretation and clinical use. Task Force of the European Society of Cardiology and the North American Society of Pacing and Electrophysiology. Circulation 1996;93:1043–65. 8598068

[37] Orini M, Bailón R, Enk R, Koelsch S, Mainardi L, Laguna P. A method for continuously assessing the autonomic response to music-induced emotions through HRV analysis. Med Biol Eng Comput 2010;48:423–33. 20300873 10.1007/s11517-010-0592-3

[38] Tarvainen MP, Niskanen J-P, Lipponen JA, Ranta-aho PO, Karjalainen PA. Kubios HRV—heart rate variability analysis software. Comput Methods Programs Biomed 2014;113:210–20. 24054542 10.1016/j.cmpb.2013.07.024

[39] Killick R, Eckley IA. Changepoint: anRPackage for changepoint analysis. J Stat Softw 2014;58:1–19.

[40] Haynes K, Killick R, Fearnhead P, Eckley I, Grose D. Changepoint.np: Methods for nonparametric changepoint detection in r. R package version 1.0.5; 2022.

[41] Soliński M, Reed CN, Chew E. A framework for modeling performers’ beat-to-beat heart intervals using music features and interpretation maps. Front Psychol 2024;15:1403599. 39295765 10.3389/fpsyg.2024.1403599 PMC11409844

[42] Gu F, Wu H. Simultaneous canonical correlation analysis with invariant canonical loadings. Behaviormetrika 2017;45:111–32.

[43] Xia CH, Ma Z, Ciric R, Gu S, Betzel RF, Kaczkurkin AN et al Linked dimensions of psychopathology and connectivity in functional brain networks. Nat Commun 2018;9:3003. 30068943 10.1038/s41467-018-05317-y PMC6070480

[44] Lynar E, Cvejic E, Schubert E, Vollmer-Conna U. The joy of heartfelt music: an examination of emotional and physiological responses. Int J Psychophysiol 2017;120:118–25. 28757232 10.1016/j.ijpsycho.2017.07.012

[45] Bak S, Shin J, Jeong J. Subdividing stress groups into eustress and distress groups using laterality index calculated from brain hemodynamic response. Biosensors (Basel) 2022;12:33. 35049661 10.3390/bios12010033 PMC8773747

[46] Gerdner LA . Effects of individualized versus classical ‘relaxation’ music on the frequency of agitation in elderly persons with Alzheimer’s disease and related disorders. Int Psychogeriatr 2000;12:49–65. 10798453 10.1017/s1041610200006190

[47] Bainbridge CM, Bertolo M, Youngers J, Atwood S, Yurdum L, Simson J et al Infants relax in response to unfamiliar foreign lullabies. Nat Hum Behav 2020;5:256–64. 33077883 10.1038/s41562-020-00963-z PMC8220405

