# Supplementary material for: Listeners’ baseline autonomic states associated with distinct music-physiology response patterns
Source: Eur Heart J Imaging Methods Pract. 2026 Jan 19;4(1):qyag013. doi: 10.1093/ehjimp/qyag013 (PMC12893215; doi:10.1093/ehjimp/qyag013)
Supplement: qyag013_Supplementary_Data [file qyag013_supplementary_data.pdf]

Listeners’ Baseline Autonomic States Associated with Distinct Music-Physiology Response Patterns

Mateusz Soliński, Vanessa Pope, Pier Lambiase, and Elaine Chew

Supplementary Materials

Table S1. The list of musical pieces included to study. Each piece was played in four versions (except Ch2, which has only two versions), one original and three with altered tempo and/or loudness. The table is based on and consistent with Table 1 in [S1].

Acronym	Performer	Composer	Piece (full name)	Versions	Version description
**Pr**	Sergei Prokofiev	Sergei Prokofiev	Gavotte op. 12 No. 2	Pr_1	original
				Pr_2	loud
				Pr_3	fast
				Pr_4	loud+fast
**SL**	Harold Bauer	Franz Schubert (transcribed by Franz Liszt)	"Ständchen" (Serenade)	SL_1	original
				SL_2	loud
				SL_3	fast
				SL_4	loud+fast
**Mo**	Wanda Landowska	Wolfgang Amadeus Mozart	Piano Sonata No. 18, K.576 (1789) (Adagio)	Mo_1	original
				Mo_2	loud
				Mo_3	fast
				Mo_4	loud+fast
**De**	Claude Debussy	Claude Debussy	La Cathédrale Engloutie (The Sunken Cathedral)	De_1	soft
				De_2	original
				De_3	fast + soft
				De_4	fast + original loudness
**Ch1**	Alfred Reisenauer	Frédéric Chopin	Berceuse Op 57 D Flat Major	$Ch1_1$	original
				$Ch1_2$	loud
				$Ch1_3$	fast
				$Ch1_4$	loud+fast
**Ch2**	Josef Hofmann	Frédéric Chopin	Nocturne in F sharp minor, Op. 48 No. 2	$Ch2_1$	original
				$Ch2_2$	fast
**Be**	Josef Lhévinne	Ludwig van Beethoven	Sonata # 14 "Moonlight" Op. 27 No. 2	Be_1	original
				Be_2	loud
				Be_3	fast
				Be_4	loud+fast
**BG**	Peter Lemer	Johann Sebastian Bach and Charles Gounod	Ave Maria (originally published in 1853 as "Méditation sur le Premier Prélude de Piano de S. Bach")	BG_1	original
				BG_2	loud
				BG_3	fast
				BG_4	loud+fast

Table S2. The number of change points in musical categories separately for each piece and version. The musical categories that were not included in the analysis are marked with grey.

	Be_1	Be_2	Be_3	Be_4	BG_1	BG_2	BG_3	BG_4	$Ch1_1$	$Ch1_2$	$Ch1_3$	$Ch1_4$	$Ch2_1$	$Ch2_2$	De_1	De_2	De_3	De_4	Mo_1	Mo_2	Mo_3	Mo_4	Pr_1	Pr_2	Pr_3	Pr_4	SL_1	SL_2	SL_3	SL_4	**Grand Total**
Tempo	3	4	2	2	9	9	2	2	7	9	7	8	5	5	9	9	3	5	6	7	2	2	5	5	3	4	5	6	5	6	**156**
Loudness	13	9	3	4	4	6	2	3	7	7	6	4	10	6	6	5	5	6	5	4	4	5	3	4	2	2	4	4	5	4	**152**
Diameter	2	2	1	1	2	2	2	2	10	8	2	3	9	4	8	8	4	3	1	2	1	0	0	0	0	0	8	9	0	0	**94**
Spectral Centroid	4	3	2	2	4	4	3	1	2	2	3	1	7	4	4	6	4	5	1	1	1	1	1	2	0	0	3	3	2	3	**79**
Significant Melody	8	6	2	1	1	1	1	1	0	0	0	0	2	2	0	1	3	1	1	4	1	1	1	0	0	0	18	8	3	4	**71**
Novel Melody	1	1	1	1	1	1	1	1	1	1	1	2	2	1	3	3	1	1	1	1	2	2	1	1	1	1	2	2	3	3	**44**
MFCC	1	2	1	1	1	2	1	0	0	1	1	1	5	1	1	3	2	2	3	3	1	0	1	0	0	0	2	1	1	1	**39**
Return	1	0	1	0	0	0	0	0	1	0	0	0	3	1	1	1	1	2	2	2	2	3	2	1	1	1	2	2	1	1	**32**
Melodic Interaction	0	0	5	1	0	0	0	0	1	0	0	1	0	0	0	0	0	0	0	0	0	0	0	2	1	2	4	4	2	2	**25**
Standout Articulation	0	0	0	1	0	0	0	0	0	0	0	0	2	0	0	0	0	0	0	0	0	0	5	1	4	6	0	4	1	1	**25**
Resolve/Release	0	1	0	0	0	1	0	0	0	0	1	1	2	1	2	1	2	1	1	1	0	0	0	0	0	0	1	1	0	2	**19**
Fast Sequence	0	0	0	0	0	0	0	0	5	3	1	3	0	0	0	0	0	0	1	1	2	2	0	0	0	0	1	0	0	0	**19**
Build-up	1	0	0	0	1	0	0	0	0	0	0	0	2	0	1	1	2	0	0	0	0	0	1	1	0	0	1	2	0	0	**13**
Climax	0	0	0	0	1	1	1	2	0	0	0	0	0	1	1	1	1	0	0	0	0	0	1	1	0	0	0	0	0	0	**11**
Emphasis	0	0	0	0	0	0	0	0	0	0	0	0	3	3	0	0	0	0	0	0	0	1	1	0	0	0	0	2	0	0	**10**
Swell	1	0	0	1	0	0	0	0	0	0	0	0	0	0	0	0	0	0	0	0	0	0	0	0	0	0	1	3	0	0	**6**
Significant Silence	0	0	0	0	1	1	1	1	0	0	0	0	0	0	0	0	0	0	0	0	0	0	0	0	0	0	0	0	0	0	**4**
Tension	0	1	0	0	2	0	0	0	0	0	0	0	0	0	0	1	0	0	0	0	0	0	0	0	0	0	0	0	0	0	**4**
Instability	0	0	0	0	0	0	0	0	1	1	2	0	0	0	0	0	0	0	0	0	0	0	0	0	0	0	0	0	0	0	**4**
Arrival	1	1	0	0	0	0	0	0	0	0	0	1	0	0	0	0	0	0	0	0	0	0	0	0	0	0	0	0	0	0	**3**
Drop / Gentle	0	0	0	0	0	0	0	0	0	0	0	0	1	1	0	0	0	0	0	0	0	0	0	0	0	0	0	0	0	0	**2**
Fast Sequence Release	0	0	0	0	0	0	0	0	0	0	0	0	0	0	0	0	0	0	0	0	0	0	0	0	0	0	0	0	1	0	**1**
**Grand Total**	**36**	**30**	**18**	**15**	**27**	**28**	**14**	**13**	**35**	**32**	**24**	**25**	**53**	**30**	**36**	**40**	**28**	**26**	**22**	**26**	**16**	**17**	**22**	**18**	**12**	**16**	**52**	**51**	**24**	**27**	**813**

Definitions of the music categories: **Novel Melody** – entrance of a prominent melody; **Return** – reappearance of a previous melodic idea; **Build-up / Significant Crescendo** – progressive increase in intensity; **Climax** – peak in musical intensity or excitement; **Resolve / Release** – transition from instability to stability; **Melodic Interaction** – interaction of two or more melodic voices; **Significant Melody** – salient or foregrounded melody; **Significant Silence** – perceptually salient pause; **Standout Articulation** – distinctive or sharply detached articulation; **Emphasis** – highlighting of a note, chord, or passage; **Runs / Fast Sequence** – rapid sequence of notes; **Swell** – gradual increase followed by decrease in intensity; **Tension** – harmonically induced sense of friction; **Instability** – perceived musical imbalance or uncertainty; **Drop / Gentle** – rapid release in intensity or dynamics; Arrival – entry into an

anticipated stable state;

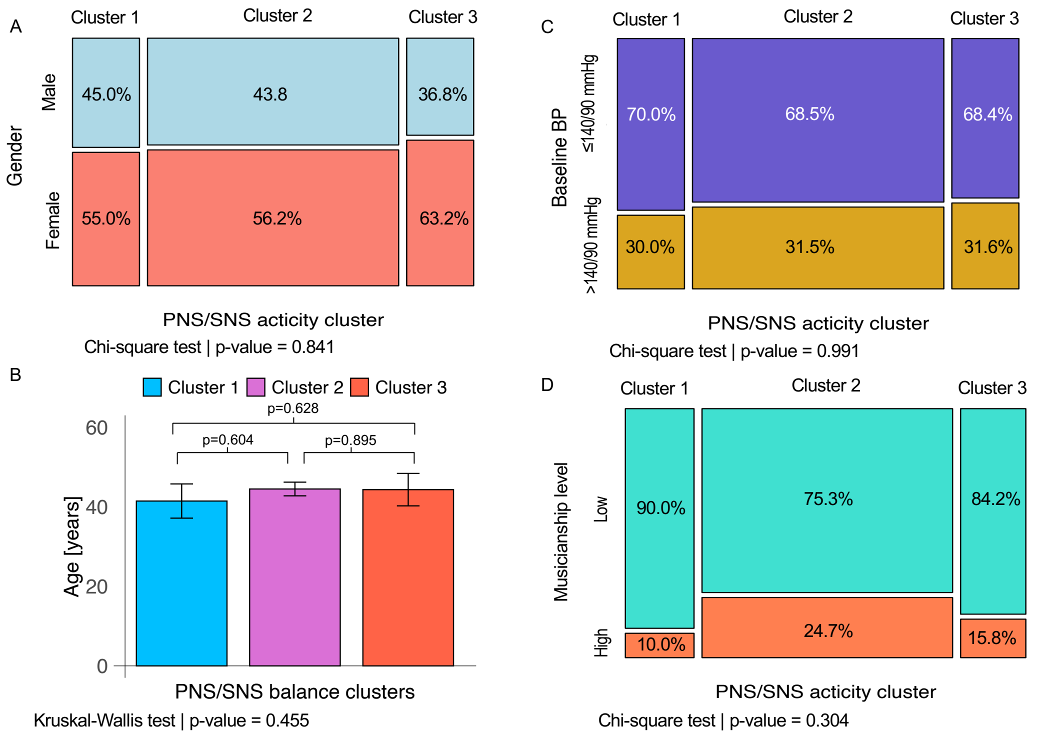

Figure S1 Comparison analysis of the gender (A), age (B), baseline BP (C), and Musicianship level (D) between clusters related to PNS/SNS balance. The results obtained from the statistical tests (chi-square and Kruskal-Wallis + Dunn Test as post-hoc test) showed no significant differences between clusters.

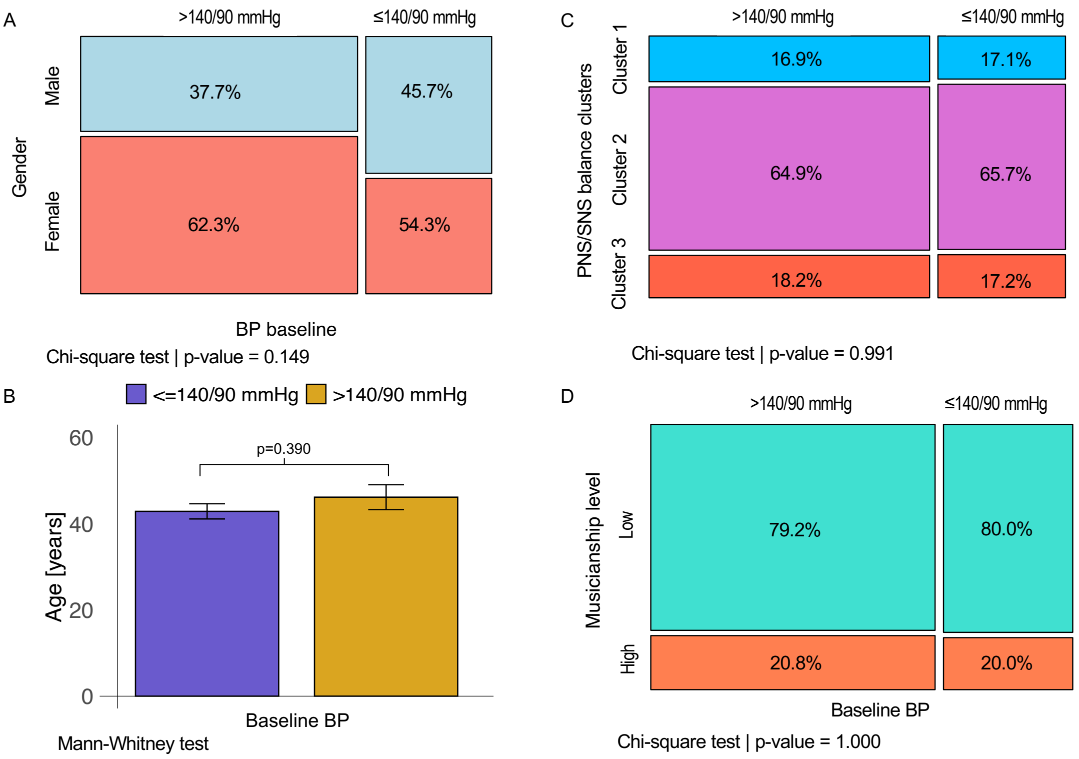

Figure S2 Comparison analysis of the gender (A), age (B), PNS/SNS balance clusters (C), and Musicianship level (D) between groups with normal and elevated baseline BP. The results obtained from the statistical tests (chi-square and Mann-Whitney) showed no significant differences between clusters.

The effect of separate pieces

The musical change points taken for the CCA analysis are from different pieces and some of them may have a differential impact on the canonical correlations between obtained variates. The number of change points for each track varies between 12 and 53 (average 27.1). Each point on the scatter plot shown in Figure S3 (A and B) is associated with a single musical change point from one of the pieces used in this study. We evaluated the effect of pieces on the values of physiological variates V_1 and V_2. The mean values and 95%CI of these variates for data points from each music piece are shown in Figure S3 (C and D). The data points whose 95%CI do not cross zero were coloured. We can observe opposite or mutual effects of some pieces on the values of the canonical variates.

In the first canonical variates, we observe that the lowest values are originated from the BG_2, BG_3(Bach's *Ave Maria*, loud and fast versions). On the other side of the scale, the largest values are observed for two pieces: Prokofiev's Gavotte (Pr_1, original version) and Schubert's Serenade (SL_4 fast+loud version).

In the second variate, data points linked with the highest values of the V_2 function are from pieces Pr_3 (fast version), Pr_1 (original version, and De_3 (Debussy's 'La Cathédrale Engloutie', fast version), and lowest De_4 (loud and fast version).

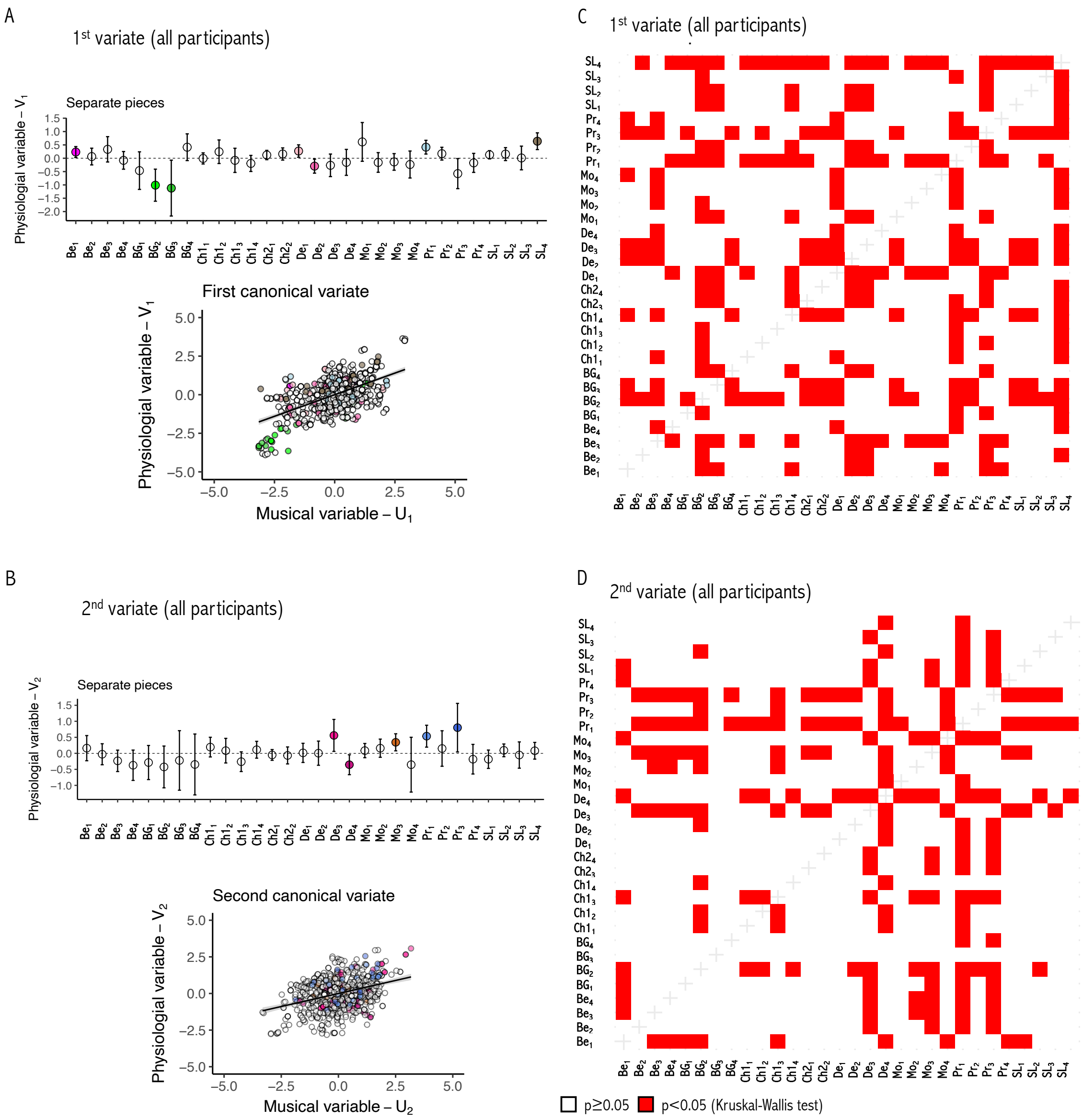

Figure S3 A-B Mean values and 95%CI of physiological variable values (V1, V2) of data points from the correlation plot (scatter plot) for each piece separately, showing the different contributions of change points to these results. Data points from the pieces whose mean values and 95%CI do not cross zero were coloured. C-D The results of the Kruskal-Wallis tests between all pairs of pieces (pairs with p-value < 0.05 were coloured with red).

Table S3. The results of difference analysis before and after the onsets of the musical change points for each physiological signal. n – number of samples (number of change points x number of participants that experienced them). The results shown the mean difference and 95% confidence intervals (in upper and lower scripts).

Category	n	Systolic BP [mmHg]	Diastolic BP [mmHg]	PP [mmHg]	Resp. Intervals [ms]	RR Intervals [ms]	P_{HF} [n.u.]	P_{LF} [n.u.]	F_{HF} [Hz]	F_{LF} [Hz]
Melodic Interaction	726	$-0.091^{0.156}_{-0.338}$	$0.076^{0.249}_{-0.097}$	$-0.167^{0.023}_{-0.311}$ *	$-26.4^{39.3}_{-92.1}$	$-0.86^{1.33}_{-3.06}$	$-0.021^{0.040}_{-0.082}$	$0.004^{0.072}_{-0.064}$	$0.003^{0.008}_{-0.001}$	$0.001^{0.003}_{-0.002}$
Novel Melody	1461	$0.118^{0.312}_{-0.076}$	$0.105^{0.249}_{-0.040}$ *	$0.013^{0.128}_{-0.101}$	$-94.5^{-39.8}_{-149.3}$ †	$1.55^{3.39}_{-0.29}$	$-0.056^{-0.007}_{-0.105}$ *	$0.044^{0.096}_{-0.007}$	$0.005^{0.009}_{0.001}$ †	$-0.006^{-0.004}_{-0.007}$ †
Resolve/Release	569	$0.098^{0.386}_{-0.189}$	$0.081^{0.304}_{-0.142}$	$0.017^{0.182}_{-0.148}$	$-12.2^{69.7}_{-94.1}$	$0.41^{3.20}_{-2.38}$	$0.055^{0.129}_{-0.019}$	$0.030^{0.109}_{-0.050}$	$-0.006^{0.000}_{-0.012}$ *	$0.002^{0.004}_{-0.001}$
Return	1136	$0.037^{0.238}_{-0.164}$	$-0.029^{0.118}_{-0.176}$	$0.067^{0.187}_{-0.054}$	$-38.9^{14.6}_{-92.4}$ *	$-1.22^{0.69}_{-3.13}$	$-0.029^{0.021}_{-0.078}$	$0.024^{0.077}_{-0.030}$	$0.003^{0.007}_{-0.001}$ *	$-0.001^{0.000}_{-0.003}$
Fast Sequence	568	$-0.173^{0.197}_{-0.543}$	$-0.062^{0.156}_{-0.279}$	$-0.034^{0.050}_{-0.117}$ *	$-18.4^{44.7}_{-81.4}$	$-1.16^{1.31}_{-3.62}$	$-0.034^{0.036}_{-0.104}$	$0.063^{0.136}_{-0.010}$	$-0.004^{0.002}_{-0.010}$	$0.001^{0.003}_{-0.002}$
Significant Melody	2301	$0.037^{0.180}_{-0.106}$	$0.071^{0.176}_{-0.034}$	$0.024^{0.117}_{-0.165}$	$4.7^{44.3}_{-34.9}$	$-0.62^{0.70}_{-1.95}$ *	$-0.010^{0.027}_{-0.046}$	$-0.039^{0.000}_{-0.078}$	$-0.002^{0.001}_{-0.005}$	$0.000^{0.002}_{-0.001}$
Standout Articulation	799	$0.029^{0.260}_{-0.201}$	$0.053^{0.227}_{-0.121}$	$0.024^{0.117}_{-0.165}$	$-55.3^{9.2}_{-119.7}$ *	$-2.02^{0.22}_{-4.26}$ *	$-0.012^{0.048}_{-0.072}$	$-0.003^{0.062}_{-0.068}$	$0.007^{0.011}_{0.002}$ *	$-0.001^{0.001}_{-0.003}$
Sp. Centroid ↓	2489	$0.132^{0.274}_{-0.009}$	$0.110^{0.213}_{0.007}$ *	$0.022^{0.107}_{-0.062}$	$-26.1^{13.9}_{-66.0}$	$0.07^{1.41}_{-1.27}$	$0.010^{0.046}_{-0.025}$	$0.010^{0.047}_{-0.026}$	$-0.001^{0.001}_{-0.004}$	$0.000^{0.001}_{-0.001}$
Sp. Centroid ↑	2369	$-0.106^{0.039}_{-0.250}$ *	$-0.009^{0.097}_{-0.115}$	$-0.097^{-0.013}_{-0.180}$ †	$-33.7^{4.0}_{-71.3}$ †	$-0.47^{0.85}_{-1.79}$ †	$-0.007^{0.032}_{-0.045}$	$0.019^{0.057}_{-0.020}$	$0.001^{0.004}_{-0.002}$	$-0.001^{0.001}_{-0.002}$
Diameter ↓	2819	$-0.062^{0.039}_{-0.250}$	$0.044^{0.142}_{-0.054}$	$-0.106^{-0.029}_{-0.183}$ †	$-20.5^{18.1}_{-59.0}$	$-0.29^{0.93}_{-1.50}$ *	$0.000^{0.033}_{-0.032}$	$0.007^{0.043}_{-0.029}$	$-0.001^{0.002}_{-0.003}$	$0.000^{0.001}_{-0.001}$
Diameter ↑	2573	$0.055^{0.190}_{-0.080}$	$0.088^{0.188}_{-0.012}$ *	$-0.033^{0.048}_{-0.114}$ *	$-9.3^{29.0}_{-47.5}$ *	$-1.83^{-0.58}_{-3.07}$ †	$-0.013^{0.020}_{-0.047}$	$-0.030^{0.008}_{-0.067}$	$0.003^{0.005}_{0.000}$ *	$-0.001^{0.000}_{-0.002}$
Loudness ↓	2081	$0.010^{0.164}_{-0.145}$	$-0.025^{0.091}_{-0.140}$	$0.034^{0.123}_{-0.055}$	$2.3^{43.4}_{-38.8}$	$-0.32^{1.18}_{-1.82}$	$0.024^{0.063}_{-0.014}$	$-0.010^{0.048}_{-0.029}$	$-0.002^{0.001}_{-0.005}$	$0.001^{0.002}_{0.000}$
Loudness ↑	2093	$0.123^{0.276}_{-0.029}$	$0.177^{0.288}_{0.066}$ *	$-0.054^{0.036}_{-0.144}$ *	$-57.9^{-17.5}_{-98.3}$ †	$-2.48^{-1.14}_{-3.81}$ †	$-0.068^{-0.030}_{-0.106}$ *	$-0.014^{0.027}_{-0.065}$	$0.006^{0.009}_{0.003}$ †	$-0.003^{-0.002}_{-0.004}$ †
MFCC ↓	1635	$0.035^{0.212}_{-0.141}$	$-0.014^{0.118}_{-0.145}$	$0.049^{0.151}_{-0.053}$	$-13.1^{34.4}_{-60.7}$	$0.037^{1.719}_{-1.645}$	$0.037^{0.081}_{-0.007}$ *	$0.061^{0.107}_{0.015}$ *	$-0.001^{0.003}_{-0.004}$	$0.000^{0.001}_{-0.002}$
MFCC ↑	2234	$0.143^{0.293}_{-0.006}$	$0.257^{0.367}_{0.147}$ †	$-0.114^{-0.025}_{-0.203}$ †	$-24.3^{14.3}_{-62.8}$ †	$-2.04^{-0.66}_{-3.43}$ †	$0.073^{0.112}_{0.034}$ *	$0.052^{0.093}_{0.012}$ *	$0.005^{0.008}_{0.002}$ †	$-0.001^{0.000}_{-0.003}$
Tempo ↓	2736	$0.065^{0.202}_{-0.073}$	$0.088^{0.189}_{-0.014}$ *	$-0.023^{0.056}_{-0.101}$	$-4.8^{34.0}_{-43.6}$	$0.45^{1.69}_{-0.80}$	$0.000^{0.033}_{-0.034}$	$0.006^{0.041}_{-0.029}$	$-0.001^{0.002}_{-0.004}$	$0.000^{0.002}_{-0.001}$
Tempo ↑	1835	$-0.035^{0.119}_{-0.189}$	$0.067^{0.182}_{-0.048}$	$-0.102^{-0.012}_{-0.192}$ *	$-55.8^{-9.8}_{-101.9}$ †	$-1.57^{-0.17}_{-2.96}$ *	$-0.070^{-0.031}_{-0.110}$ †	$-0.025^{0.018}_{-0.067}$	$0.006^{0.010}_{0.003}$ †	$-0.002^{-0.001}_{-0.003}$ *

* $p<0.05$, † significant difference after using Bonferroni correction ($p<0.00033$)

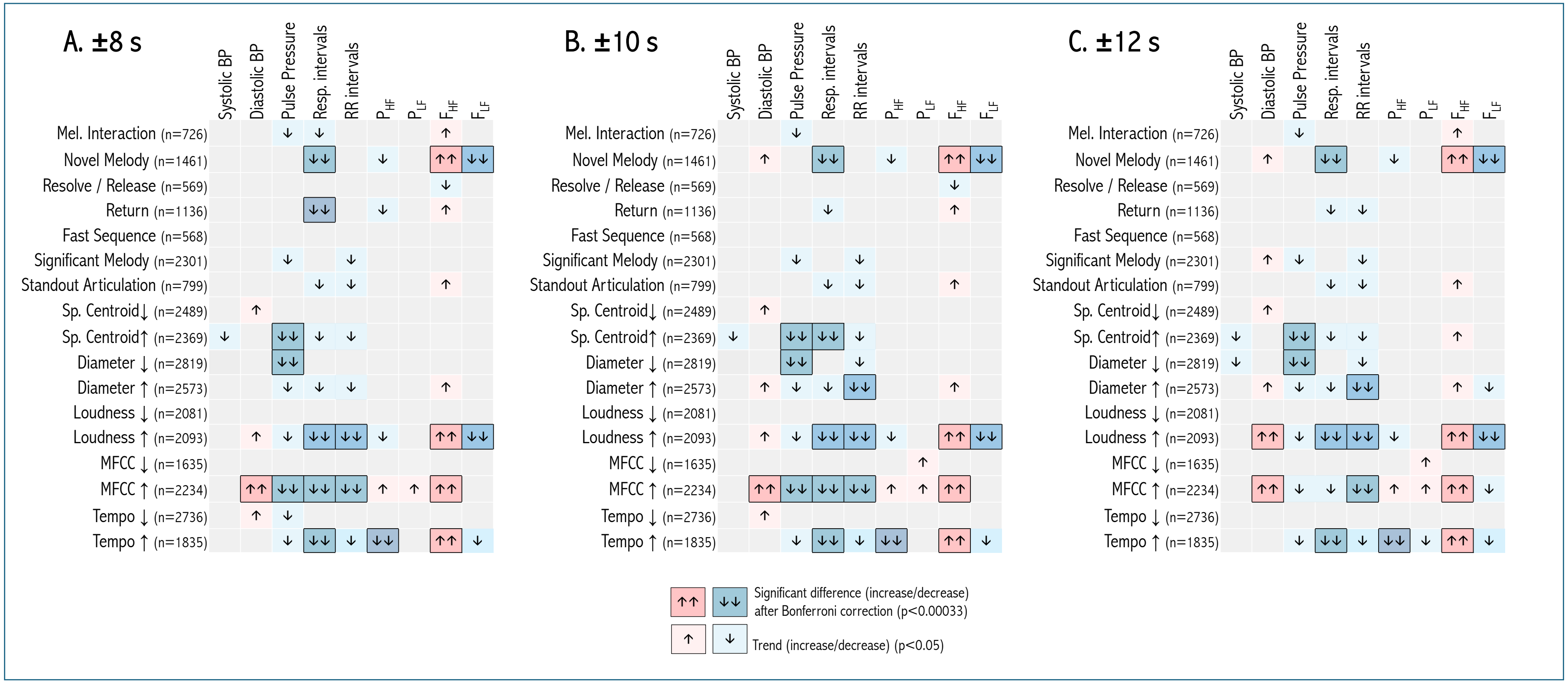

Figure S4 A 10 s window was chosen as a compromise that provides sufficient support for interval-based measures (RR and respiratory intervals and derived autonomic indices) and beat-to-beat blood pressure, while remaining temporally local to individual musical events. The shorter windows (5 s) would contain too few samples (before interpolation), especially for lower heart and respiratory rates, to maintain methodological relevance. In turn, longer windows (15–20 s) substantially increase overlap with neighbouring change points, especially in expressive music, confounding attribution. As an additional sanity validation for the chosen window length, we performed the analysis of the statistical results on differences between before and after the onsets of music change points, considering A. 8 seconds, B. 10 seconds, and C. 12 seconds before and after the onset of the change points. The results show modest stability in the signs of the differences and in the p-values indicating significant change.

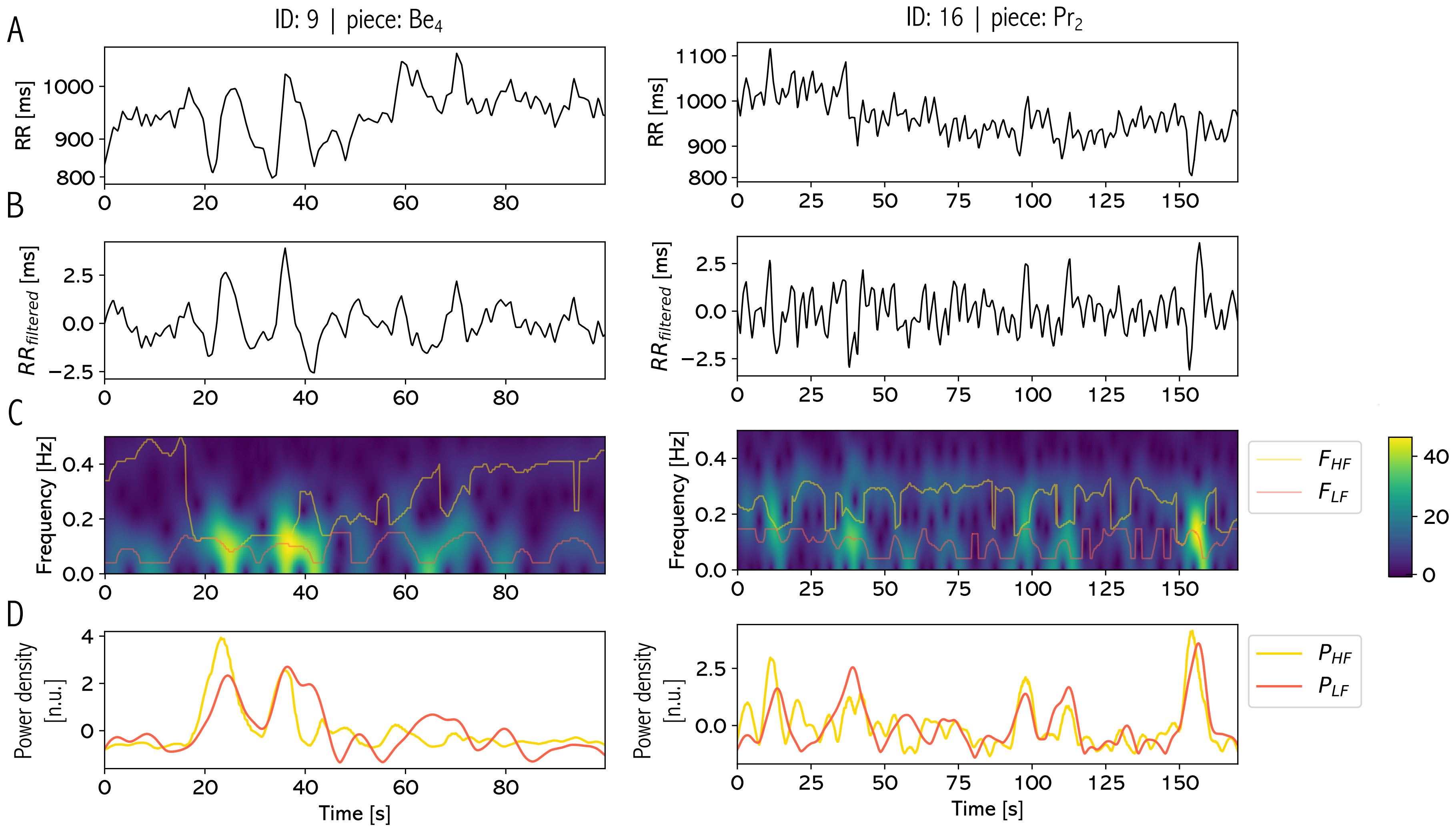

Figure S5 Two examples of signals used for calculating HRV spectral parameters P_{HF}, P_{LF}, F_{HF}, F_{LF}, according to the method described by Orini et al. (Orini et al. 2010). **A** RR interval series from two participants (ID9 and ID16), **B** Filtered RR interval series (high-pass Butterworth filter with a cut-off frequency of 0.03 Hz). **C** Spectrograms based on the short-time Fourier transform (STFT). The instantaneous peak frequencies in LF and HF bands (F_{LF}, F_{HF}) in each second were marked by lines. **D** Time series of power density in LF and HF bands (P_{LF}, PHF) extracted from spectrograms.

Table S4. The number of participants provided different answers about formal training in music theory and the daily practice of a musical instrument. We classify the participants according to their responses to the statements: "I engaged in regular, daily practice of a musical instrument (including voice) for X years" and "I have had formal training in music theory for X years". A high musicianship level was assigned to people with at least 6 years of regular, daily practice and 3 years of formal music theory training, respectively. The criteria for musicianship were similar to the ones proposed by the Burunat et al. study [S2]. The cluster, considered a high level of musicianship, was coloured purple (otherwise green).

High musicianship level			I have had formal training in music theory for X years							
Low musicianship level		0	0.5	1	2	3	3-4	4-6	>7	Σ
I engaged in regular, daily practice of a musical instrument (including voice) for X years	0	26	1	1	2	1	1	1	1	34
	1	4	1	1	1	0	0	1	0	8
	2	3	1	1	1	0	0	0	0	6
	3	7	0	1	3	0	0	0	1	12
	3-4	2	1	0	0	1	0	0	0	4
	4-5	3	0	1	1	0	0	1	0	6
	6-9	4	0	3	2	3	0	3	2	17
	>10	3	1	1	4	2	1	5	8	25
	Σ	52	5	9	14	7	2	11	12	112

Gaussian Kernel Functions

The first step was to create binary vectors for each musical and physiological signal with a length equal to the piece's duration. The onset of each change point (rounded to an integer value) was marked with value 1 in these vectors; otherwise, the vectors were filled with zeros.

$$b_i = \begin{cases} 1, & \text{if there is a change point at time } i \\ 0, & \text{otherwise} \end{cases}$$

For change points detected automatically on musical signals, the onset annotations were multiplied by the difference between the mean values of the signal taken from the signal's excerpts before and after the change point (the borders of the excerpts are two consecutive change points). This approach introduces weights to the analysis, assigning greater importance to the change points with larger changes in mean and minimising the impact of the change points related to small changes in signals.

$$b_i^* = \begin{cases} \Delta\mu_i, & \text{if } b_i = 1 \\ 0, & \text{otherwise} \end{cases}$$

$$\Delta\mu_i = \mu_{\text{after}} - \mu_{\text{before}}$$

where μ_{before} and μ_{before} are the mean values of the signal in the segments before and after t_i, respectively.

A GKF is applied at each change point in the binary vector to smooth its representation in time. The resulting time series $g(t)$ is created by adding Gaussian kernel centred at each i where $b_i^* \neq 0$:

$$g(t) = \sum_{i=0}^{T} b_i^* \cdot \frac{1}{\sigma\sqrt{2\pi}} \exp\left(-\frac{(t-i)^2}{2\sigma^2}\right)$$

where $\sigma = 10$ seconds. This value was selected to approximate the individual response delay to each detected change point. We validated the results for the wide range of σ between 1 and 20 seconds to check the stability of observed effects. The results for the entire study cohort are presented in Figure S9. We found that the canonical loadings remained consistent across the full range of σ values. Additionally, the canonical correlations for the first variate increase with σ, although this growth stabilised around $\sigma = 10$ seconds. For further variates, canonical correlation coefficients plateaued around this threshold.

For physiological signals, the final Gaussian kernel time series $G(t)$ is obtained by averaging the individual Gaussian kernel series $g^{(p)}(t)$ over all participants P who listened to the piece:

$$G(t) = \frac{1}{P}\sum_{p=1}^{P} g^{(p)}(t)$$

The resultant Gaussian kernel density functions for each musical piece reflect the distribution of change points in the musical and physiological signals. An example of the functions of a selected piece is shown in Figure 1 in the main article.

The input for the CCA analysis was extracted from the time series with Gaussian kernel functions described before. For each change point among all musical categories, two vectors:

$$\mathbf{x}_i = [x_1(t_i), x_2(t_i), \dots, x_n(t_i)]$$
$$\mathbf{y}_i = [y_1(t_i), y_2(t_i), \dots, y_m(t_i)]$$

were created, where the variables x and y relate to the time series from the musical and physiological datasets, respectively. The resultant input data are two matrices X and Y aggregating vectors $\mathbf{x}_i$ and $\mathbf{y}_i$ from all $i = 1,2,\dots,T$ change points:

$$\mathbf{X} = (\mathbf{x}_{t_1} \quad \mathbf{x}_{t_2} \quad \cdots \quad \mathbf{x}_{t_T})$$
$$\mathbf{Y} = (\mathbf{y}_{t_1} \quad \mathbf{y}_{t_2} \quad \cdots \quad \mathbf{y}_{t_T})$$

These matrices **X** and **Y** contain the values from the all-time series at all musical change points.

SIGNIFICANCE TESTING OF CANONICAL VARIETIES

Wilks' Lambda test is used to assess the overall significance of the canonical correlation by considering all pairs of canonical variates in a hierarchical scheme; the tests verify the null hypothesis that there are no significant differences between groups on a combination of variables used to perform CCA. First, the full model (variates from 1 to 9) is tested, delivering general Wilks' λ (which is equivalent to $1 - R^2$, where R^2 is determination coefficient for the model). Then, variates from 2 to 9 and the other hierarchies are tested until the last variate is tested by itself. The Wilks' Lambda was approximated using the F distribution that allows the calculation

of p-values for each hierarchy (the significance was considered for $p < 0.05$. Note that the final variates in the canonical analysis are usually weak and uninterpretable, but due to the large data size, they might be still significant; they often indicate small, non-meaningful effects, which raises the risk of overinterpreting the results [S5]. Therefore, the Wilks' Lambda test might be insufficient for verifying the significance of the effect observed in the latter canonical variates. Some other precautions in selecting the final set of variables to analyse are needed.

The process of the final selection of the variates was enhanced by the surrogate data analysis. Complementary datasets X_{surr} and Y_{surr} were created based on randomly selected time stamps instead of the time onsets of musical change points. The mean values and standard deviations of canonical correlations for each variate were calculated on 100 repetitions of these datasets. The canonical variates from the original data can be further interpreted if their canonical correlation coefficients are higher than the highest canonical correlation in the surrogate datasets (or the mean value from the highest coefficients in the analysis of clusters).

An additional criterion was a noticeably high covariance value (compared to the rest of the variates) calculated as the percentage of information explained by the canonical variate observed in a scree plot. A common (but often problematic) method is to look at the point where the values start decreasing significantly slower (i.e., the 'elbow'). However, in the current dataset, such changes were usually difficult to notice. Moreover, we assessed the orthogonality of the effects in the successive canonical variates and the practical significance of the findings.

The final criterion was to obtain consistent and comparable results between the different participant groups. Based on the preliminary results, we decided to report the effects observed in the first two variates in all groups of participants considered. In some cases, a third variate could also be considered, but this was usually on the threshold of meeting the above-mentioned criteria.

Permutation testing

We used a permutation testing procedure as an additional test of the statistical significance of the canonical variates selected for interpretation. The permutation was performed by shuffling the matrix rows containing physiological variables (**Y**), whereas the matrix with musical ones (**X**) remained unchanged. The resampling procedure was repeated 1000 times, and the distributions of the canonical correlations were obtained for each variate. We calculated the number of correlation values from these distributions, comparing them to the correlations estimated on the original dataset (P_{perm}). We found that the original correlations in selected variates were higher than the permuted ones in all considered groups of participants (whole population, PNS-SNS clustering, baseline BP levels, musicianship level). The distributions and P_{perm} are presented in *Figures S6-S8*.

Permutation tests

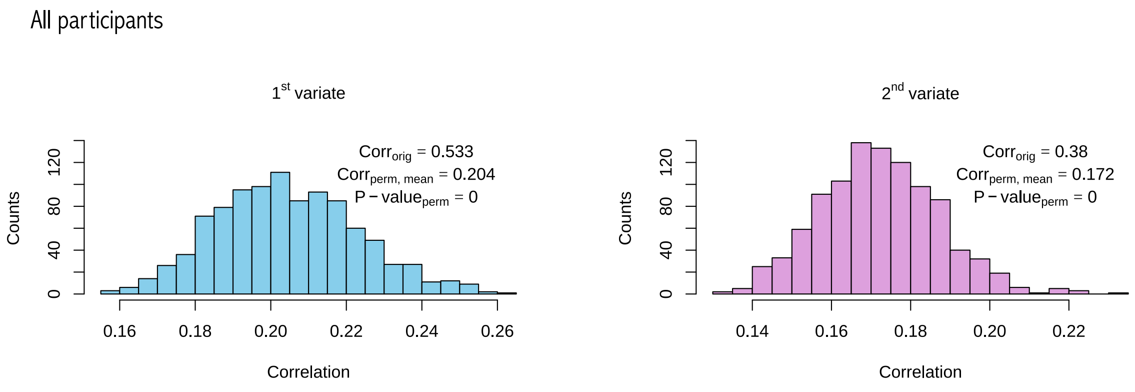

Figure S6 Distributions of the correlation values of the first two canonical variates obtained from the 1000 permutations of the entire data set.

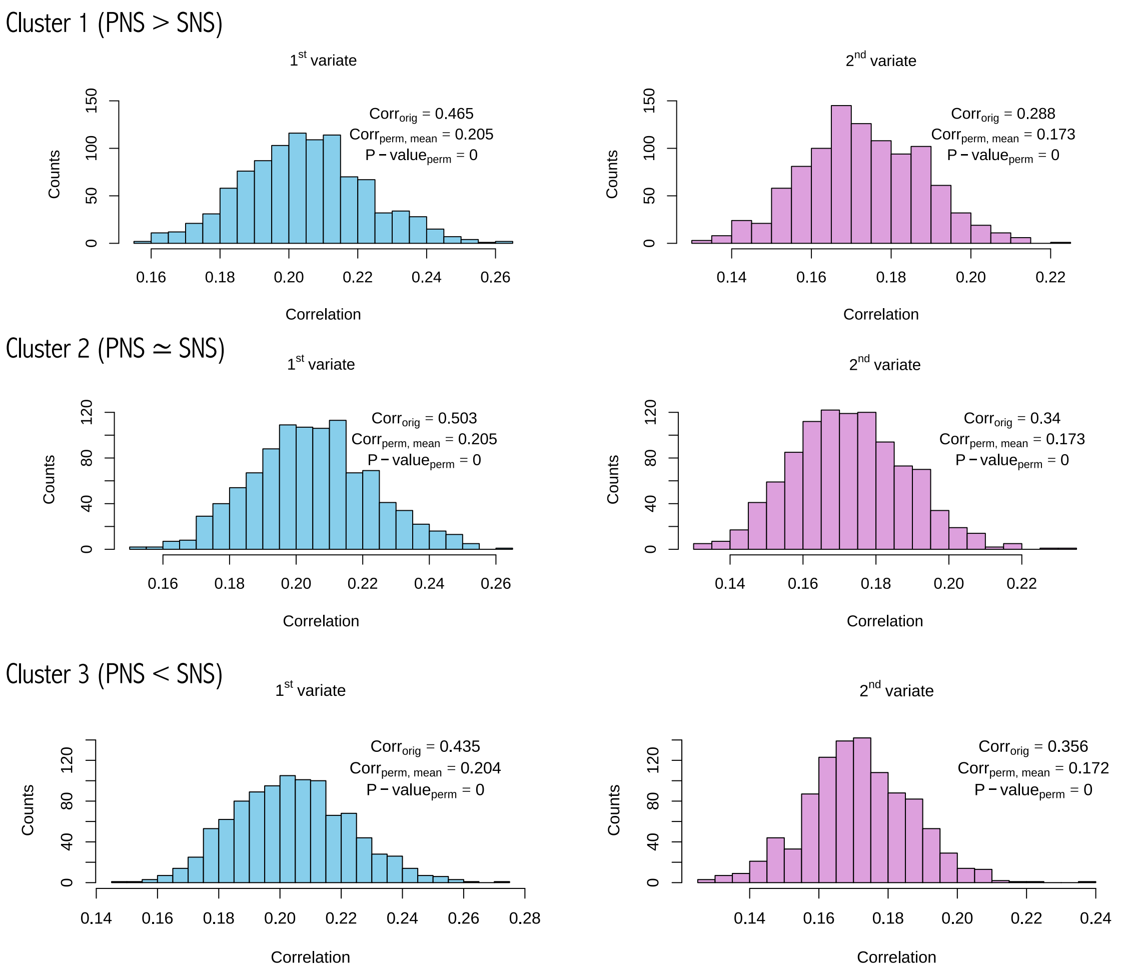

Figure S7 Distributions of the correlation values of the first two canonical variates obtained from the 1000 permutations of subsets of data (clusters stratified by the levels of PNS and SNS activity).

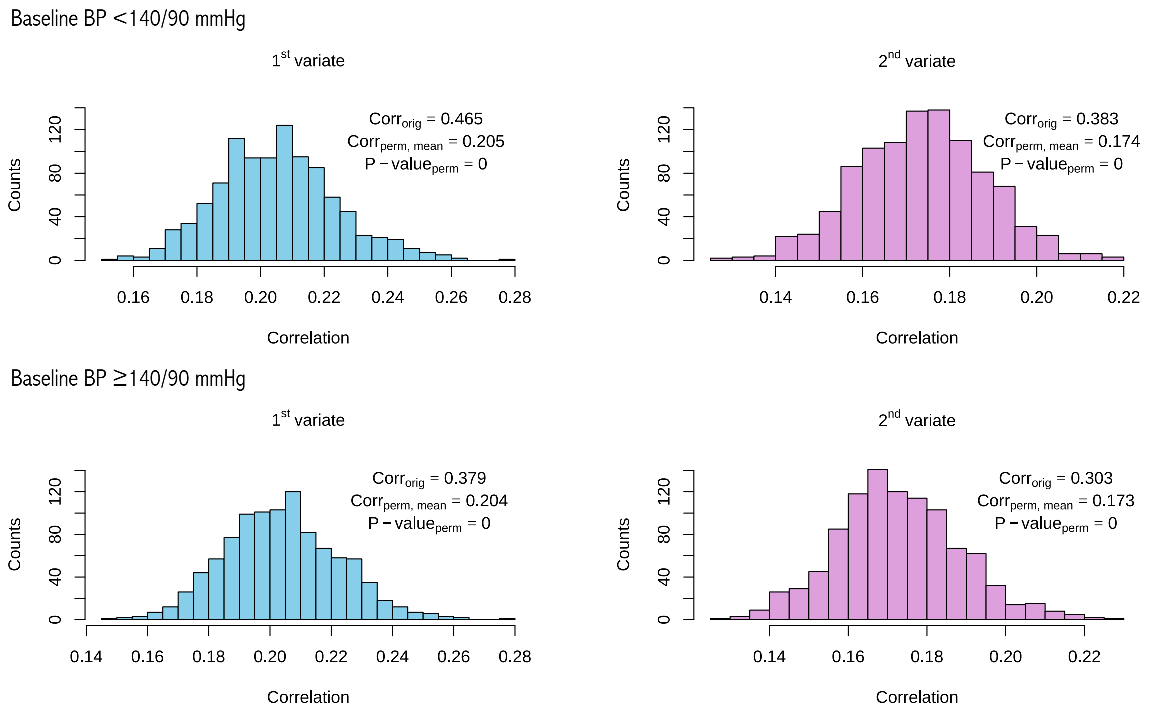

Figure S8 Distributions of the correlation values of the first two canonical variates obtained from the 1000 permutations of subsets of data (based on the level of the baseline BP).

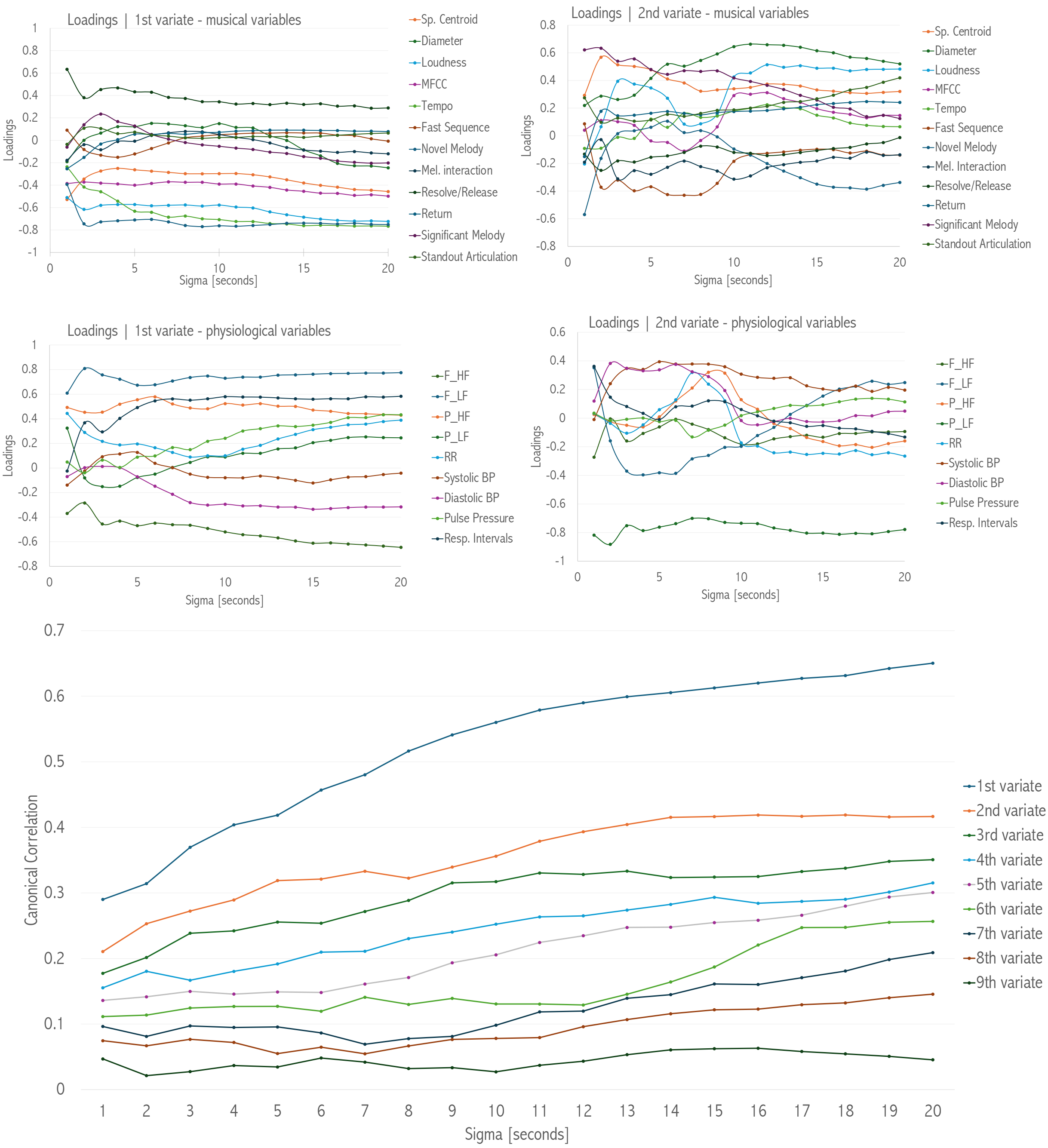

Figure S9. The stability analysis of the loading values for different values of the sigma parameter (standard deviation of the Gaussian Kernel Function). The results shown that the loading values remain stable in a broad range of sigma.

Generalised Additive Models for analysis of the Effect of Age, Gender, Personal Musical Characteristics on music-physiology patterns

We investigated the effect of baseline physiological features (baseline SNS/PNS index values, baseline systolic/diastolic BP) and inter-participant differences (age, gender, musicianship level, and the preference for classical music) on shaping the music-physiology patterns observed in CCA results for the whole dataset. To do so, we performed CCA for each individual and measured the similarity (or dissimilarity) of the loadings obtained (from the first variate) with the ones calculated for the entire dataset. Then, we used generalised additive models (GAMs) to calculate the association between baseline and clinical features with the similarity values:

$$Similarity_i \sim \beta_0 + f_1(Age_i) + f_2(Sys_i) + f_3(Dia_i) + f_4(PNS_i) + f_5(SNS_i) + \beta_1 Gender_i + \beta_2 Musician_i + \beta_3 FavClassical_i + \varepsilon_i$$

where f_1 to f_5 are smooth splines estimated using cubic splines (df = 4),

We used Generalised Additive Models (GAMs) to evaluate the similarity differences between listeners regarding their baseline physiological indices (SNS/PNS indexes, systolic/diastolic blood pressure), and demography (age, gender, musicianship level, and music preference).

Generalised Additive Models (GAMs) extend Generalised Linear Models (GLMs) by allowing for flexible, non-linear relationships between predictors and the response variable. This is achieved by replacing fixed linear terms with smooth functions–typically splines–while preserving the additive structure of the model. The similarity between individual and group-level loading vectors was quantified using cosine similarity. Alternative metrics, including Pearson correlation, dot product, and Euclidean distance, were also evaluated, with largely consistent results across methods, except in the case of Euclidean distance. Unlike Euclidean distance, which emphasises absolute differences in magnitude, cosine similarity captures directional alignment between vectors. This makes it particularly well-suited for comparing loading patterns, where the relative contribution of features is more informative than their absolute scale. The analysis using GAMs yields significant estimates (p-values) for each spline and the partial effects associated with the variables.

Table S5. The results of the Generalised Additive Models (GAM) of the similarity between individual and group-level canonical loadings, considering baseline characteristics and inter-subject differences.

	Coef.	Std.Err.	z	P>|z|	[0.025	0.975]
**Intercept**	0.247	0.145	1.701	0.089	-0.038	0.531
**bs(age, df=4)**	-0.345	0.238	-1.451	0.147	-0.812	0.121
**bs(age, df=4)**	0.096	0.219	0.439	0.660	-0.333	0.526
**bs(age, df=4)**	-0.541	0.308	-1.757	0.079	-1.144	0.063
**bs(age, df=4)**	0.078	0.205	0.380	0.704	-0.323	0.479
**Intercept**	0.247	0.145	1.701	0.089	-0.038	0.531
**bs(NBP_sys, df=4)**	-0.220	0.263	-0.836	0.403	-0.737	0.296
**bs(NBP_sys, df=4)**	0.125	0.301	0.416	0.678	-0.465	0.715
**bs(NBP_sys, df=4)**	-0.502	0.347	-1.450	0.147	-1.182	0.177
**bs(NBP_sys, df=4)**	0.326	0.298	1.093	0.275	-0.259	0.910
**Intercept**	0.247	0.145	1.701	0.089	-0.038	0.531
**bs(NBP_dia, df=4)**	0.147	0.423	0.349	0.727	-0.681	0.975
**bs(NBP_dia, df=4)**	0.088	0.366	0.240	0.810	-0.629	0.804
**bs(NBP_dia, df=4)**	0.053	0.435	0.123	0.902	-0.800	0.907
**bs(NBP_dia, df=4)**	-0.413	0.382	-1.083	0.279	-1.161	0.335
**Intercept**	0.247	0.145	1.701	0.089	-0.038	0.531
**bs(PNS, df=4)**	-0.942	0.460	-2.051	**0.040**	-1.843	-0.042
**bs(PNS, df=4)**	-0.654	0.498	-1.314	0.189	-1.630	0.322
**bs(PNS, df=4)**	-0.598	0.562	-1.065	0.287	-1.699	0.503
**bs(PNS, df=4)**	-0.552	0.650	-0.848	0.396	-1.826	0.723
**Intercept**	0.247	0.145	1.701	0.089	-0.038	0.531
**bs(SNS, df=4)**	-0.025	0.478	-0.052	0.958	-0.961	0.912
**bs(SNS, df=4)**	0.756	0.694	1.090	0.276	-0.604	2.115
**bs(SNS, df=4)**	-1.145	0.784	-1.459	0.144	-2.682	0.393
**bs(SNS, df=4)**	-0.348	0.705	-0.493	0.622	-1.730	1.035
**Gender (binary)**	-0.133	0.061	-2.201	**0.028**	-0.252	-0.015
**Musicianship Level (binary)**	-0.023	0.073	-0.313	0.754	-0.166	0.120
**Classical Music Preference (binary)**	-0.097	0.071	-1.364	0.172	-0.237	0.042

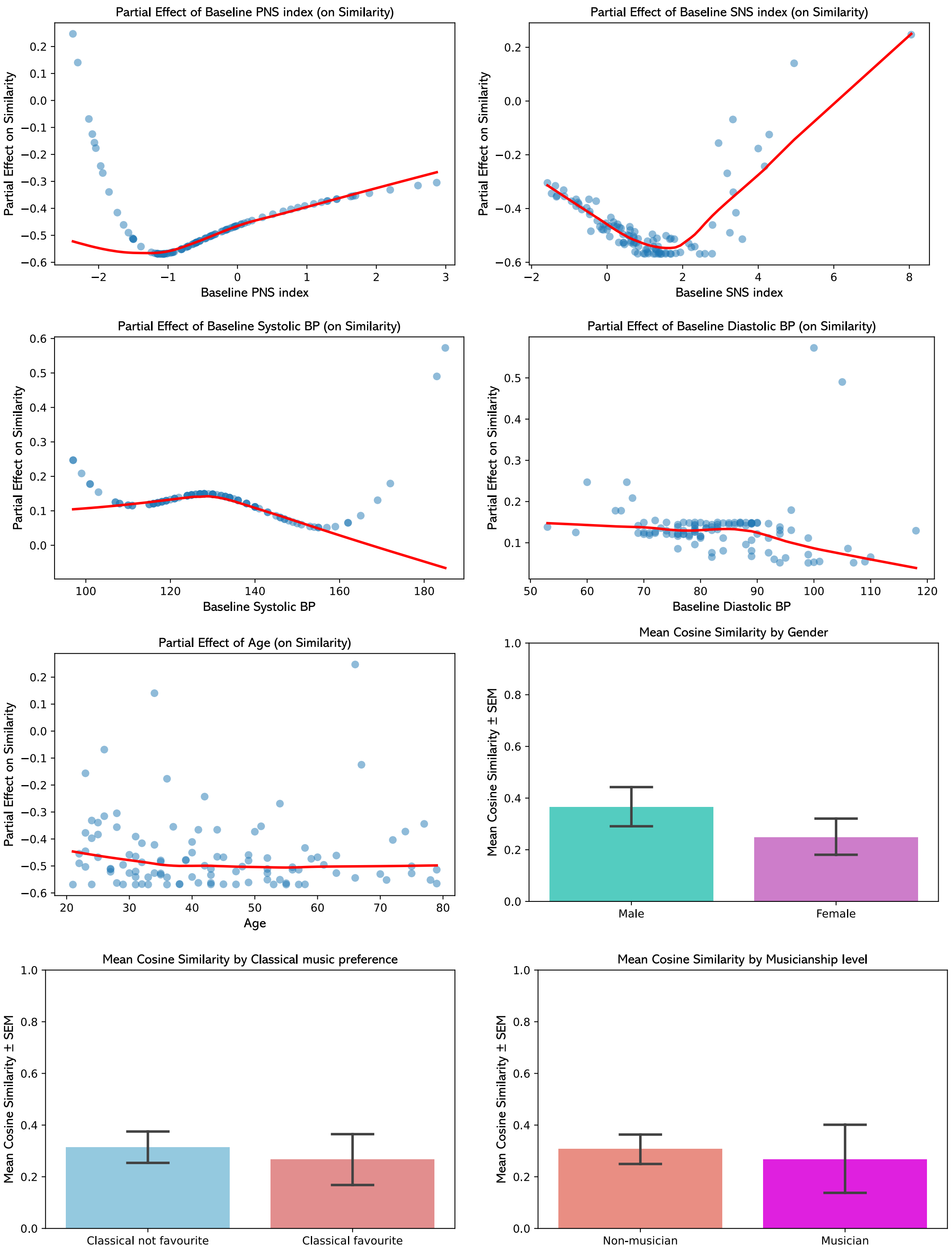

Figure S10. Partial effects for continuous and discrete variables used in the GAMs framework.

CHANGE POINT DETECTION

Both approaches define a change point when in an ordered sequence of data, $y_{1:n} = (y_1, .., y_n)$ there exists a time $\tau \in 1, ..., n-1$, for which the statistical properties (for example, mean) of $y_1, ... y_\tau$ and $y_{\tau+1}, ... y_n$ are different in some way.

The detection of a single change point can be formulated as a hypothesis testing problem. The null hypothesis (H_0) assumes that no change point is present (m = 0), while the alternative hypothesis (H_1) posits the existence of a single change point (m = 1) that splits the signal into two segments (m+1). The verification of these hypotheses is based on estimating the likelihood of a change in mean. The function $cpt.mean()$ estimates the likelihood with the assumption that signal samples are normally distributed [S3], while the function $cpt.np()$ uses an empirically determined distribution (more details about this method can be found in [S4]).

The likelihood test statistic can be extended to multiple change points (m>1) by summing the likelihood for each of the segments. The problem becomes one of minimising a cost function (based on negative log-likelihood) over all possible combinations of $\tau_{1:m}$:

$$\sum_{i=1}^{m+1} [C(y_{(\tau_{i-1}+1):\tau_i})] + \beta f(m),$$

where C is a cost function and $\beta f(m)$ is a penalty against over-fitting. The minimisation of cost functions requires verification of the null hypotheses over many combinations of change points by using iterative algorithms differentiated by the computational time and required assumptions. In case of normally distributed signals and $cpt.mean()$ function, two algorithms were considered: BinSeg (binary segmentation) and PELT (pruned exact linear time). For other distributions (and $cpt.np()$ function), ED-PELT (PELT with a cost based on the empirical distribution; [S3]). Another aspect concerning most methods for determining change points is the condition that successive sample signals should be independent of each other. This assumption is naturally not fulfilled for most physiological signals (and some musical), as autocorrelation is usually observed. The framework for change point detection that integrates the aforementioned approaches is presented schematically in Figure S11.

The first step involves assessing whether the signal follows a normal distribution using the Shapiro–Wilk test. Based on the test outcome, one of two functions is selected for change point detection: $cpt.mean()$ for normally distributed data, or $cpt.np()$ from the $changepoint.np$ package for non-normal distributions. This procedure is repeated across 100 randomly selected configurations of input parameters, including data normalisation, sampling frequency for interpolation, the maximum number of change points, penalty values, p-value threshold for the Shapiro–Wilk test, and the cost function minimisation method (these parameters were found as important in obtaining differential detection of change points). The list of configurations is shown in Table S6 in the Supplementary Materials. Detected change point indices are then aggregated across all iterations. Note, that we chose to use 100 randomly sampled configurations, rather than an exhaustive parameter grid, after observing that increasing the number of combinations did not improve detection quality and instead introduced additional noise into the aggregated results.

The next step is to calculate the probability of finding a change point in each second of the signal. It is calculated by dividing the number of detected change points at a given second (N_{chp}^i) by the total number of iterations from the previous step. The local change point is considered meaningful if its probability is larger than 0.5 or N_{chp}^i is larger than the 95th percentile of $[N_{chp}]$ (distribution of numbers of detected change points at each second). Additionally, we introduced a

minimum distance of 5 seconds between the final change points for all time series, except for respiratory intervals, systolic blood pressure (BP), diastolic BP, and pulse pressure (PP), for which it was set to 10 seconds due to their lower original sampling frequency (and consequently, slower changes). The examples of the change point detection are shown in Figures S12 and S13.

Change points detection framework

This framework is designed to identify the most meaningful change points in time series data by performing numerous iterations of change point detection under varying input parameters.

For each time series—whether musical or physiological—change points were detected using cpt.mean() (Killick et al., 2014) for normally distributed data and changepoint.np() (Haynes et al., 2022) for non-normal distributions. Each detection function was applied across 100 randomly selected combinations of input parameters. These repeated detections were used to estimate the change point probability P^{i}_{chp} at each second, which was then used to determine the final set of change point indices.

This probabilistic, ensemble-based approach increases robustness by minimizing the influence of specific parameter choices, making it especially well-suited for complex or noisy time series where true change points may be subtle or context-dependent.

Combinations of change point detection functions parameters:

- signal normalisation (yes/no)
- sample frequency for interpolation (1 Hz, 5 Hz, 10 Hz)
- SW test: p-value threshold (0.001 or 0.05)
- maximum number of change points (only for cpt.mean: (5, 10, 20)
- penalty value for cpt.mean (2·log(n), 4·log(n), 8·log(n), 16·log(n), 32·log(n))
- penalty value for changepoint.np (2·log(n), 4·log(n), 8·log(n), 16·log(n), 32·log(n))
- detection method (cpt.mean): (BinSeg, PELT)

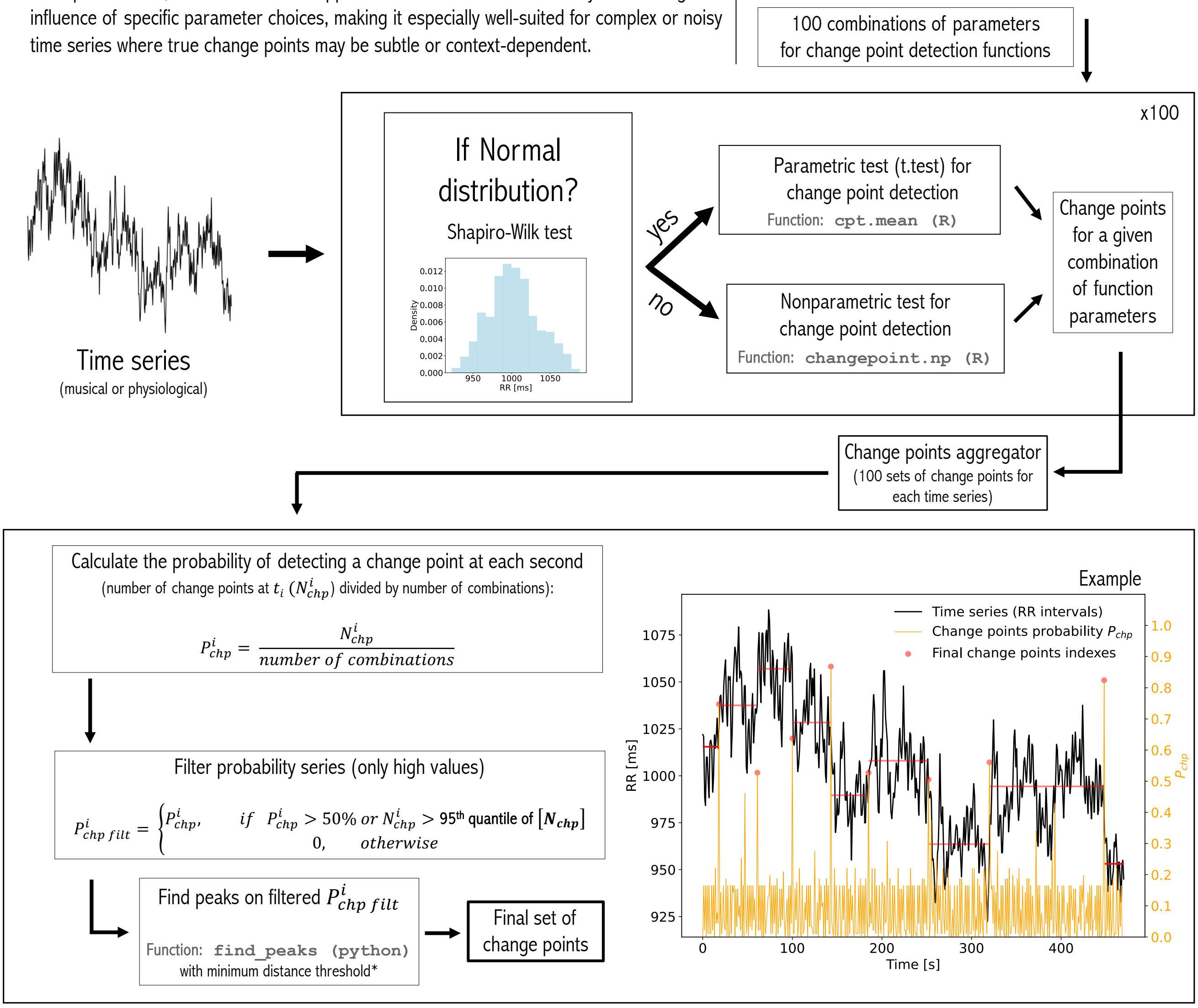

* The minimum distance between change points was set to 5 seconds for all time series, except for respiratory intervals, systolic blood pressure (BP), diastolic BP, and pulse pressure (PP), for which it was set to 10 seconds due to their lower original sampling frequency (and consequently, slower changes).

(Killick et al., 2014) Killick R, Eckley IA. "changepoint: An R package for changepoint analysis". Journal of statistical software. 2014 Jun 25;58:1-9.

(Haynes et al., 2022) Haynes K, Killick R, et al. "changepoint.np: Methods for Nonparametric Changepoint Detection" (R package library), CRAN: Contributed Packages, Jul 2022, version 1.0.5.

Figure S11. A diagram depicting the framework for detecting change points.

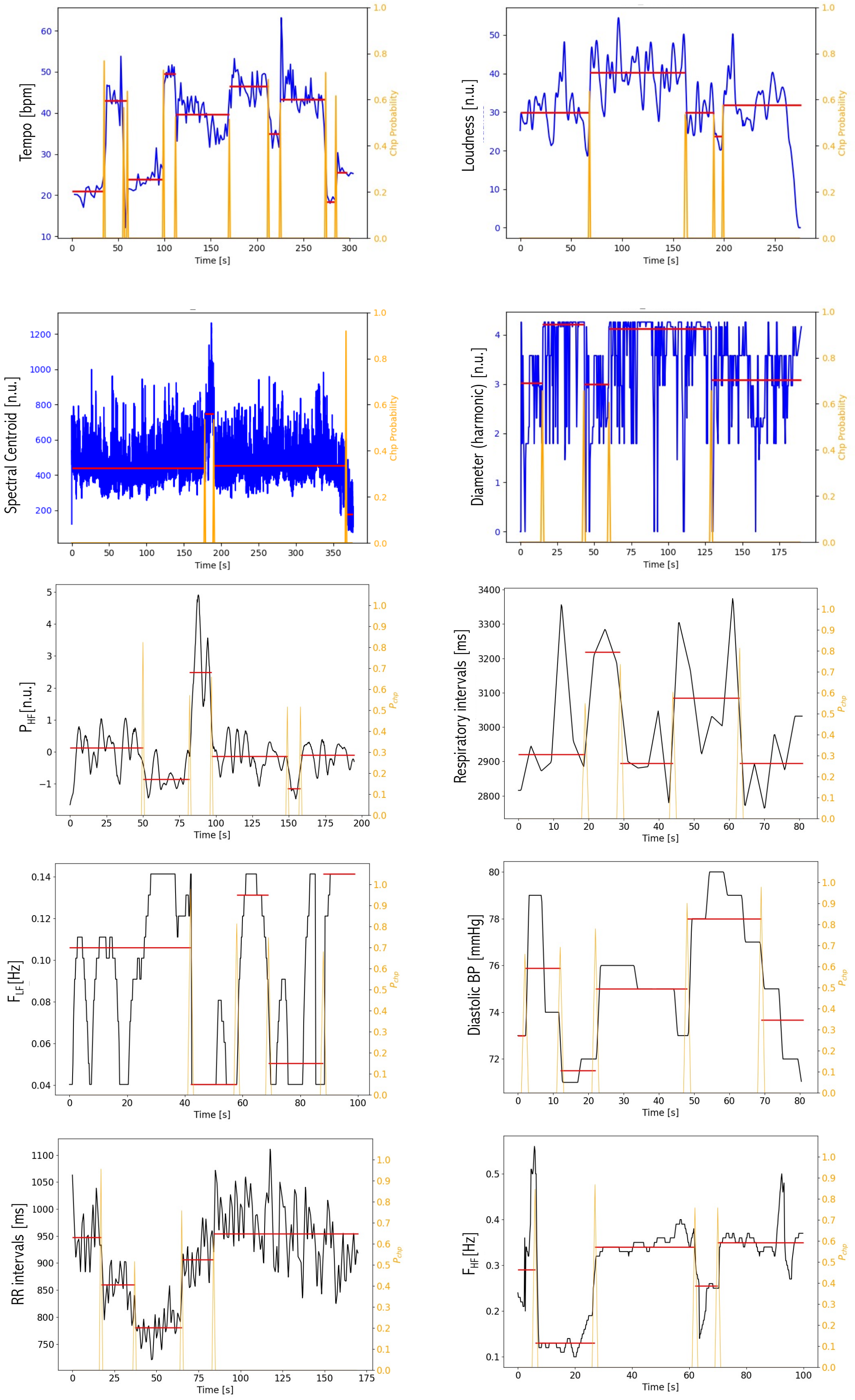

Figure S12. Example change point detections in different signals (musical and physiological).

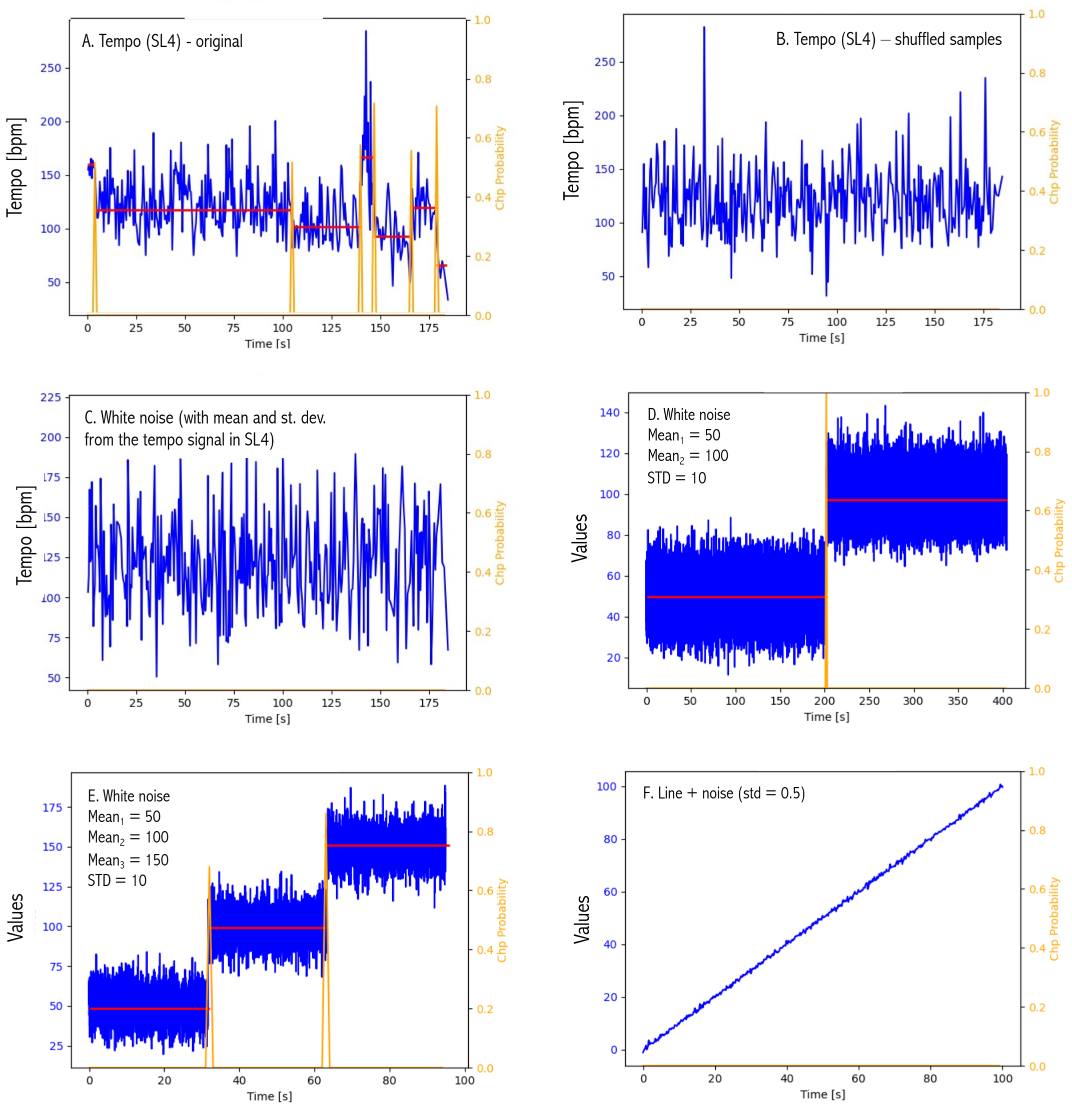

Figure S13. Example change point detections across different synthetic signals. A. Original signal (tempo, piece: SL4) with change points detected; B. a tempo signal (from A) with shuffled samples, which destroyed the original dynamics and no change points are expected; C. A random noise signal with the same mean and standard deviation as in A; D. A random noise signal with two means and constant standard dev. (one change point expected); E. A random noise signal with three means and a constant standard deviation. (two change points expected); F. A monotonously increasing signal (a line) with added random noise (no change points expected).

Table S6. The list of 100 combinations of parameters used in the change point detection framework.

Iteration	Signal Normalisation	Sample frequency for signals interpolation	Max number of change points	penalty value (cpt.mean)	Algorithm (cpt.mean)	penalty value cpt.mean	normality thershold - SW test
0	TRUE	10	10	32*log(n)	BinSeg	2*log(n)	0.05
1	FALSE	5	20	8*log(n)	PELT	2*log(n)	0.05
2	TRUE	1	20	16*log(n)	PELT	2*log(n)	0.001
3	FALSE	1	5	32*log(n)	PELT	8*log(n)	0.001
4	FALSE	10	5	2*log(n)	BinSeg	32*log(n)	0.05
5	TRUE	10	10	8*log(n)	BinSeg	32*log(n)	0.05
6	FALSE	1	10	4*log(n)	BinSeg	2*log(n)	0.05
7	FALSE	10	5	8*log(n)	BinSeg	32*log(n)	0.05
8	TRUE	5	10	8*log(n)	PELT	16*log(n)	0.001
9	FALSE	1	5	16*log(n)	BinSeg	16*log(n)	0.05
10	FALSE	1	5	16*log(n)	PELT	8*log(n)	0.001
11	FALSE	1	20	4*log(n)	PELT	8*log(n)	0.001
12	FALSE	5	20	2*log(n)	PELT	2*log(n)	0.05
13	TRUE	10	5	2*log(n)	BinSeg	4*log(n)	0.001
14	TRUE	1	10	16*log(n)	PELT	2*log(n)	0.001
15	TRUE	5	10	4*log(n)	BinSeg	32*log(n)	0.001
16	TRUE	5	10	32*log(n)	BinSeg	32*log(n)	0.05
17	FALSE	10	10	8*log(n)	BinSeg	8*log(n)	0.05
18	TRUE	1	10	16*log(n)	PELT	32*log(n)	0.001
19	TRUE	5	10	8*log(n)	PELT	4*log(n)	0.05
20	FALSE	10	5	32*log(n)	PELT	8*log(n)	0.05
21	FALSE	10	10	8*log(n)	PELT	32*log(n)	0.05
22	TRUE	1	5	16*log(n)	PELT	32*log(n)	0.001
23	TRUE	5	5	32*log(n)	PELT	4*log(n)	0.05
24	TRUE	5	20	2*log(n)	PELT	4*log(n)	0.001
25	TRUE	5	10	8*log(n)	BinSeg	32*log(n)	0.05
26	TRUE	5	5	2*log(n)	BinSeg	16*log(n)	0.05
27	TRUE	10	5	4*log(n)	PELT	32*log(n)	0.001
28	TRUE	10	10	4*log(n)	BinSeg	2*log(n)	0.001
29	FALSE	1	5	32*log(n)	PELT	2*log(n)	0.05
30	FALSE	5	5	2*log(n)	PELT	4*log(n)	0.001
31	TRUE	1	10	16*log(n)	BinSeg	8*log(n)	0.001
32	FALSE	10	20	8*log(n)	PELT	32*log(n)	0.001
33	FALSE	10	5	32*log(n)	PELT	8*log(n)	0.001
34	TRUE	1	5	2*log(n)	PELT	2*log(n)	0.05
35	TRUE	10	20	8*log(n)	BinSeg	4*log(n)	0.001

36	FALSE	1	5	16*log(n)	PELT	16*log(n)	0.05
37	TRUE	10	10	2*log(n)	PELT	2*log(n)	0.05
38	TRUE	10	10	16*log(n)	PELT	32*log(n)	0.001
39	TRUE	10	10	2*log(n)	BinSeg	32*log(n)	0.05
40	TRUE	10	20	4*log(n)	BinSeg	8*log(n)	0.001
41	FALSE	1	20	8*log(n)	PELT	2*log(n)	0.05
42	TRUE	10	5	8*log(n)	BinSeg	8*log(n)	0.001
43	FALSE	1	20	8*log(n)	PELT	8*log(n)	0.05
44	TRUE	1	5	2*log(n)	BinSeg	8*log(n)	0.05
45	FALSE	1	5	16*log(n)	PELT	2*log(n)	0.001
46	TRUE	1	10	2*log(n)	BinSeg	16*log(n)	0.05
47	TRUE	10	5	32*log(n)	PELT	8*log(n)	0.001
48	FALSE	1	5	16*log(n)	PELT	16*log(n)	0.001
49	TRUE	5	20	4*log(n)	PELT	8*log(n)	0.001
50	TRUE	5	20	8*log(n)	BinSeg	2*log(n)	0.001
51	TRUE	10	10	16*log(n)	PELT	16*log(n)	0.05
52	FALSE	10	20	4*log(n)	BinSeg	4*log(n)	0.001
53	TRUE	1	10	16*log(n)	BinSeg	16*log(n)	0.05
54	FALSE	1	20	8*log(n)	BinSeg	16*log(n)	0.05
55	TRUE	1	20	8*log(n)	PELT	8*log(n)	0.001
56	FALSE	1	20	16*log(n)	PELT	16*log(n)	0.001
57	TRUE	10	20	4*log(n)	PELT	8*log(n)	0.05
58	FALSE	10	10	16*log(n)	BinSeg	4*log(n)	0.001
59	FALSE	10	20	8*log(n)	PELT	2*log(n)	0.001
60	TRUE	5	10	32*log(n)	PELT	2*log(n)	0.001
61	FALSE	1	10	32*log(n)	PELT	16*log(n)	0.05
62	FALSE	10	20	16*log(n)	BinSeg	4*log(n)	0.001
63	FALSE	10	10	8*log(n)	PELT	32*log(n)	0.001
64	TRUE	1	20	8*log(n)	PELT	32*log(n)	0.001
65	TRUE	1	5	4*log(n)	PELT	2*log(n)	0.001
66	FALSE	1	5	8*log(n)	PELT	8*log(n)	0.001
67	TRUE	5	10	32*log(n)	BinSeg	8*log(n)	0.05
68	FALSE	10	20	16*log(n)	BinSeg	32*log(n)	0.001
69	FALSE	5	10	32*log(n)	BinSeg	16*log(n)	0.05
70	TRUE	5	20	16*log(n)	BinSeg	32*log(n)	0.001
71	TRUE	5	10	16*log(n)	BinSeg	32*log(n)	0.001
72	TRUE	10	5	16*log(n)	PELT	2*log(n)	0.05
73	FALSE	10	20	4*log(n)	BinSeg	16*log(n)	0.05
74	FALSE	10	10	8*log(n)	BinSeg	32*log(n)	0.001
75	FALSE	10	10	32*log(n)	PELT	8*log(n)	0.05
76	TRUE	10	20	4*log(n)	PELT	32*log(n)	0.05
77	FALSE	5	10	2*log(n)	BinSeg	8*log(n)	0.05

78	TRUE	5	20	2*log(n)	BinSeg	32*log(n)	0.05
79	FALSE	10	10	32*log(n)	BinSeg	4*log(n)	0.001
80	TRUE	10	5	4*log(n)	PELT	32*log(n)	0.05
81	FALSE	5	20	4*log(n)	BinSeg	8*log(n)	0.001
82	TRUE	1	20	32*log(n)	BinSeg	4*log(n)	0.001
83	TRUE	1	20	4*log(n)	BinSeg	32*log(n)	0.05
84	FALSE	10	20	32*log(n)	BinSeg	2*log(n)	0.05
85	TRUE	10	5	8*log(n)	PELT	32*log(n)	0.05
86	TRUE	5	5	4*log(n)	BinSeg	4*log(n)	0.05
87	TRUE	1	10	8*log(n)	BinSeg	2*log(n)	0.001
88	FALSE	1	10	8*log(n)	PELT	8*log(n)	0.05
89	FALSE	5	5	16*log(n)	PELT	8*log(n)	0.05
90	FALSE	5	5	8*log(n)	PELT	8*log(n)	0.05
91	TRUE	10	20	8*log(n)	BinSeg	2*log(n)	0.001
92	FALSE	10	5	4*log(n)	PELT	2*log(n)	0.001
93	TRUE	10	10	2*log(n)	BinSeg	8*log(n)	0.05
94	FALSE	10	5	2*log(n)	PELT	32*log(n)	0.001
95	FALSE	1	10	8*log(n)	BinSeg	32*log(n)	0.05
96	TRUE	10	10	8*log(n)	PELT	16*log(n)	0.001
97	TRUE	5	5	16*log(n)	BinSeg	32*log(n)	0.001
98	TRUE	5	20	2*log(n)	BinSeg	8*log(n)	0.001
99	FALSE	10	5	2*log(n)	PELT	2*log(n)	0.001

EXPLORATORY SUBJECT-WISE CCA ANALYSIS

EXPLORATORY SUBJECT-WISE CANONICAL CORRELATION ANALYSIS.

To further assess individual-level variability in music–physiology coupling, we performed canonical correlation analysis separately for each participant using their own set of musical and physiological change-point responses. For each subject, we extracted music–physiology interaction terms defined as the product of musical and physiological loadings. Because dominant associations may appear in either the first or second canonical variate, interactions were evaluated across both variates, and for each subject the interaction with the larger absolute magnitude was retained.

Subject-wise canonical solutions were aligned to the group-level solution to resolve sign indeterminacy. For selected music–physiology pairs identified as prominent in the group-level analysis, we quantified the proportion of participants whose interaction direction matched the group-level coupling direction. Deviations from an equal (50/50) distribution of aligned versus opposite signs were assessed using χ^2 goodness-of-fit tests.

This analysis revealed substantial inter-individual heterogeneity in interaction magnitudes, while several dominant music–physiology couplings exhibited non-random directional tendencies across participants (Table S7), supporting the group-level interpretation while explicitly acknowledging individual variability.

Table S7. Directional alignment of individual music–physiology couplings relative to the group-level CCA solution

**Pair (music × physiology)**	**Negative sign (n, %)**	**Positive sign (n, %)**	**χ^2 (df = 1)**	**p-value**
Novel × F_LF	75 (70.1%)	32 (29.9%)	17.28	0.00003
Novel × Resp	62 (57.9%)	45 (42.1%)	2.7	0.1
Tempo × F_LF	74 (69.2%)	33 (30.8%)	15.71	0.00007
Tempo × Resp	68 (63.6%)	39 (36.4%)	7.86	0.005
Loudness × RR	72 (67.3%)	35 (32.7%)	12.79	0.00035
Loudness × P_HF	58 (54.2%)	49 (45.8%)	0.76	0.38

Selected cluster-comparison tests for key response measures around major musical event categories

To provide complementary context for the multivariate findings, we present selected before–after analyses of physiological responses around major musical event categories, stratified by autonomic cluster. These analyses were conducted to examine whether event-locked physiological changes exhibit consistent directional trends or differences across clusters and to assess how cluster-level response patterns relate to the multivariate associations identified by canonical correlation analysis. The results are intended as descriptive sanity checks rather than as primary inferential tests.

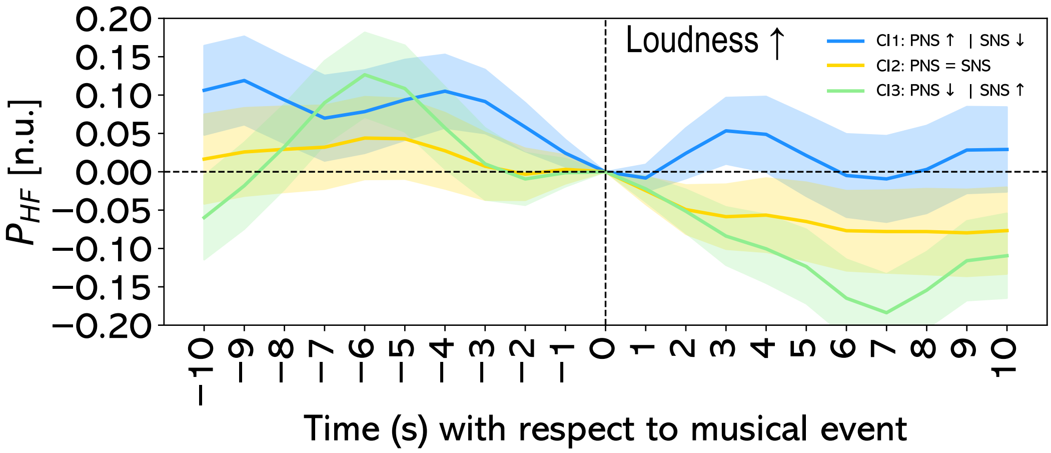

Change point category	cluster	Mean Δ P_{HF} [n.u.]	N of events	pvalue
Loudness ↑	CL1	-0.065 (-0.158 - 0.027)	353	0.168
	CL2	-0.087 (-0.139 - -0.035)	1237	<0.001
	CL3	-0.145 (-0.24 - -0.049)	350	0.008

Figure S14. Table S8. Changes in P_{HF} before and after the change point associated with an increase in loudness. A significant decrease in P_{HF} observed in cluster 3 is consistent with high absolute loading values for this cluster in the first canonical variate ($P_{HF} = -0.66$; Loudness = 0.75).

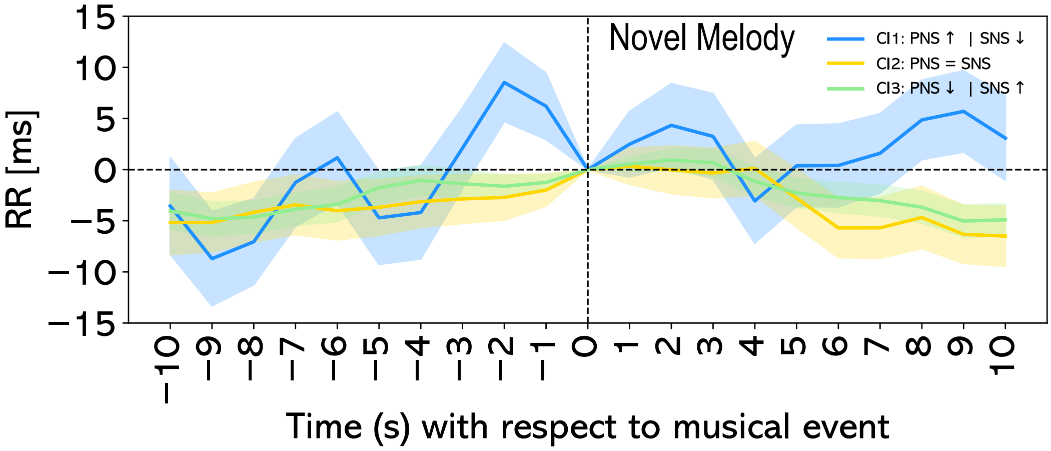

Change point category	cluster	Mean Δ RR [ms]	N of events	pvalue
Novel Melody	CL1	3.451 (-2.914 - 9.817)	208	0.137
	CL2	0.478 (-2.129 - 3.085)	752	0.628
	CL3	0.719 (-2.273 - 3.712)	206	0.803

Figure S15. Table S9. Changes in RR before and after the change point associated with a Novel Melody. No significant differences observed; however, an increase in RR after Novel Melody is coherent with loading values for cluster 1 in the first canonical variate for these categories (RR = 0.61; Novel Melody = 0.76).

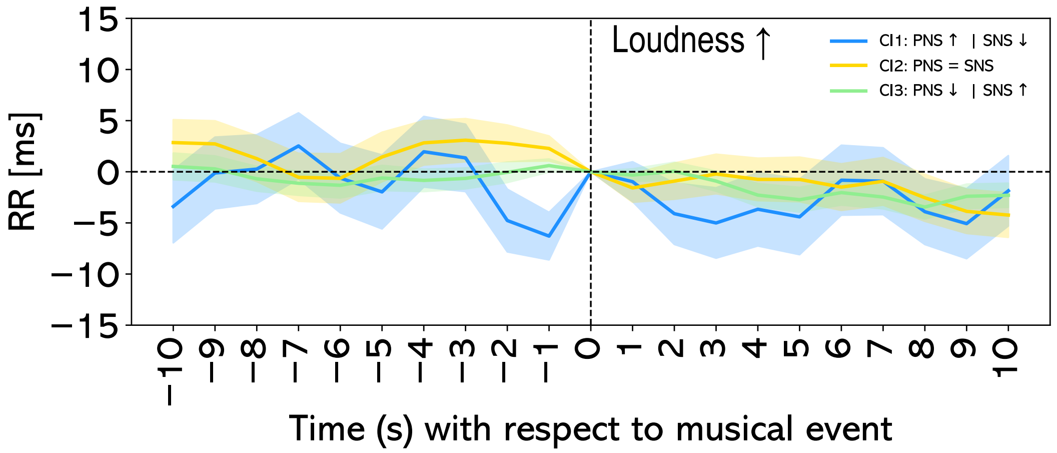

Change point category	cluster	Mean ∆ RR [ms]	N of events	pvalue
Loudness ↑	CL1	-1.968 (-6.511 - 2.575)	353	0.129
	CL2	-3.54 (-5.305 - -1.774)	1243	0.000
	CL3	-1.497 (-3.49 - 0.497)	350	0.025

Figure S16. Table S10. Changes in RR before and after the change point associated with an increase in loudness. A significant decrease in RR intervals observed in cluster 3 is consistent with high absolute loading values for this cluster in the first canonical variate (RR = −0.63; Loudness = 0.75).

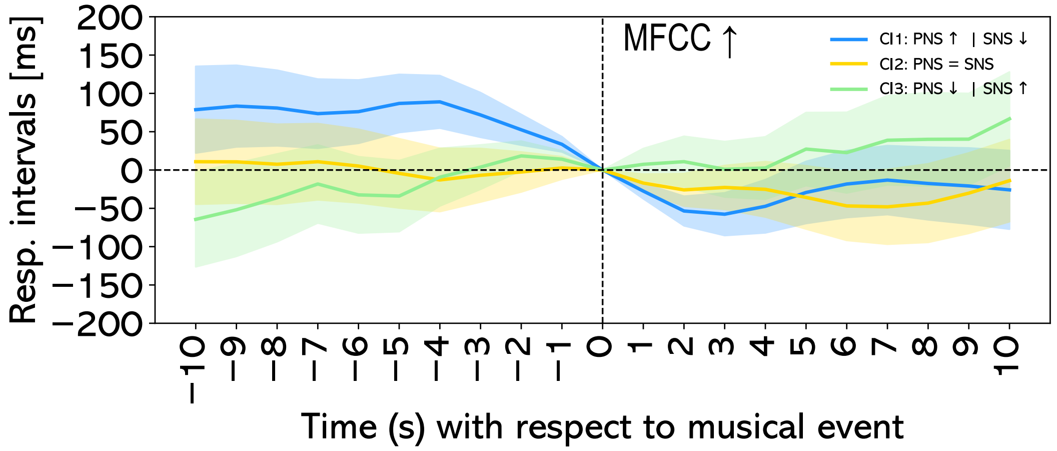

Change point category	cluster	Mean ∆ Resp. Int. [ms]	N of events	pvalue
MFCC ↑	CL1	-103.8 (-197.4 - -10.1)	346	0.003
	CL2	-33.0 (-86.6 - 20.6)	1206	0.019
	CL3	46.8 (-81.1 - 174.8)	342	0.179

Figure S17. Table S11. Changes in Resp. intervals before and after the change point associated with MFCC ↑. A significant decrease in the intervals observed in cluster 1 is consistent with high absolute loading values for this cluster in the first canonical variate (Resp. Int. = −0.63; MFCC = 0.49).

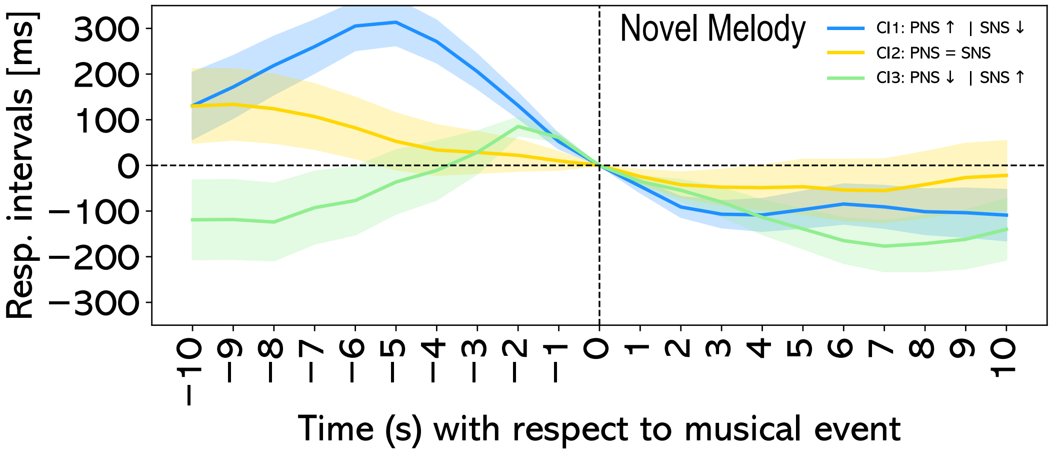

Change point category	cluster	Mean Δ Resp. Int. [ms]	N of events	pvalue
Novel Melody	CL1	-299.9 (-426.4 - -173.3)	208	<0.001
	CL2	-113.3 (-197.5 - -29.1)	746	<0.001
	CL3	-83.4 (-240.7 - 73.9)	206	0.006

Figure S18. Table S12. Changes in Resp. Intervals before and after the change point associated with a Novel Melody. Significant differences were observed for all clusters with the largest for cluster 1, which is consistent with high absolute loading values for this cluster in the first canonical variate (Resp. Int. = −0.63; Novel Melody = 0.76).

References

[S1] Pope VC, Soliński M, Lambiase PD, Chew E. High blood pressure inhibits cardiovascular responsiveness to expressive classical music. Scientific Reports. 2025 Mar 29;15(1):10908.

[S2] Burunat I, Levitin DJ, Toiviainen P. Breaking (musical) boundaries by investigating brain dynamics of event segmentation during real-life music-listening. Proceedings of the National Academy of Sciences. 2024 Sep 3;121(36):e2319459121.

[S3] Killick R, Eckley IA. changepoint: An R package for changepoint analysis. Journal of statistical software. 2014 Jun 25;58:1-9.

[S4] Haynes K, Killick R, Fearnhead P, Eckley I, Grose D. changepoint. np: Methods for nonparametric changepoint detection. R package version 0.0. 2016;2.

[S5] Sherry A, Henson RK. Conducting and interpreting canonical correlation analysis in personality research: A user-friendly primer. Journal of personality assessment. 2005 Feb 1;84(1):37-48.
